# Toward Chromatography-Free Convergent Peptide Synthesis via Nanostar Sieving

**DOI:** 10.1021/acs.oprd.6c00123

**Published:** 2026-08-17

**Authors:** Mustafa Al-Kadhimi, James Botwright, Adam Oxley, Seok Ju Han, Nil Tandogan Gray, Michael O. Frederick, Ludmila Peeva, Andrew G. Livingston

**Affiliations:** † School of Engineering and Materials Science, 4617 Queen Mary University of London Joseph Priestley Building, Mile End Road, London E1 4NS, U.K.; ‡ Exactmer Ltd, Londoneast-UK Business and Technical Park, Yew Tree Avenue, London, Dagenham RM10 7FN, United States; § Synthetic Molecule Design and Development Eli Lilly and Company Lilly Corporate Centre, Indianapolis, Indiana 46285, United States

**Keywords:** liquid-phase peptide synthesis (LPPS), fragment condensation, chromatography-free purification, nanostar sieving, organic solvent nanofiltration (OSN), membrane-assisted purification

## Abstract

Liquid-phase peptide synthesis (LPPS) has emerged as a versatile and efficient method for assembling peptides, offering significant advantages in purity and yield. In this study, we investigate the application of organic solvent nanofiltration (OSN) technology in LPPS for peptide fragment assembly by Nanostar Sieving. We report how a nanostar support decorated with solubilizing polymers can be used to achieve solubility at concentrations of up to 40 mM of a 39-mer peptide. This is assembled from eight peptide fragments on an 8-arm, 20 kDa nanostar support. OSN enables effective removal of reagents, byproducts, and unreacted fragments after each coupling cycle, enhancing synthesis efficiency. A photolabile linker was incorporated into the support before fragment assembly, enabling periodic analysis without full global deprotection. The Rink amide linker facilitated the final cleavage of the synthesized peptide using standard global deprotection protocols. Following synthesis, OSN was employed to isolate the peptide from the support. In contrast to previous OSN-assisted peptide fragment assembly methods, which required chromatographic purification to remove unreacted fragments at the end of synthesis, nanostar sieving enables their removal during synthesis. The potential to eliminate the need for chromatography could lead to more efficient and scalable therapeutic peptide production.

## Graphical abstract

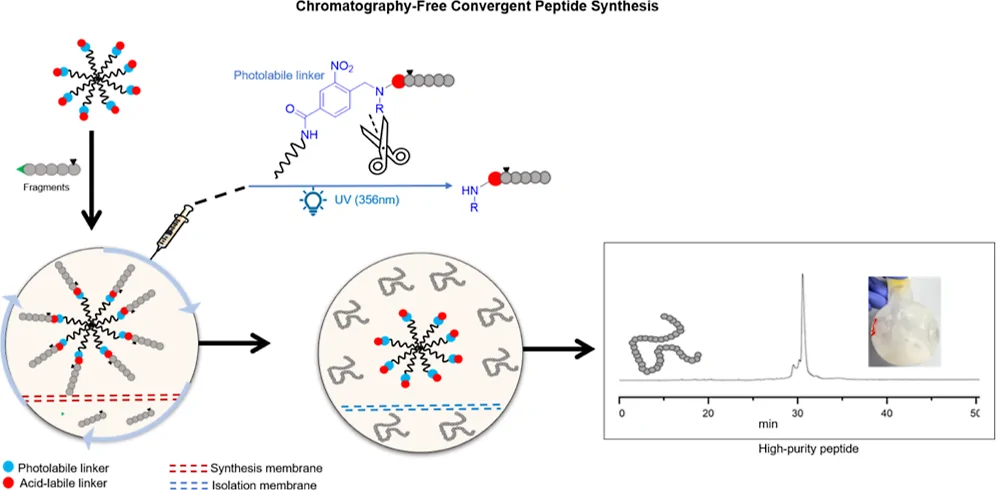

## Introduction

Over the past two decades, the therapeutic potential of peptides has driven pharmaceutical companies to invest heavily in peptide-based drug discovery and development programs.^1^ The majority of these peptides are used in the treatment of cancer, diabetes, obesity, cardiovascular diseases, and gastrointestinal disorders.^2,3^ Additionally, the urgent need to find alternatives to classical antibiotics, due to the growing threat of antibiotic resistance, has led to a rapid increase in research on antimicrobial peptides (AMPs).^4^ More recently, the Covid-19 pandemic has further accelerated efforts to develop novel therapeutic peptides to combat SARS-CoV-2 infection and related diseases.^5^

Peptides play essential roles in biological processes, including enzymatic activity, signal transduction, and cellular communication. Due to their significance, peptides have become indispensable in drug development, medical diagnostics, and biomedical imaging.^6^ The therapeutic potential of peptides has been underscored by the recent regulatory approval of blockbuster peptide drugs such as semaglutide (Ozempic/Wegovy) and tirzepatide (Mounjaro/Zepbound), driving increased interest in peptide-based therapeutics across both scientific and public domains.^7–12^ Consequently, the development of efficient, scalable peptide synthesis methods is of paramount importance to the pharmaceutical and biomedical sectors.

In liquid-phase peptide synthesis (LPPS), peptides are synthesized in solution, which allows for reaction kinetics more similar to conventional solution-phase chemistry. By incorporating a support that imparts distinct physicochemical properties (e.g., hydrophobicity or increased molecular size), selective separation of the growing peptide from unreacted reagents and byproducts can be achieved. Several LPPS strategies have been reported, often employing precipitation or extraction for purification.^13–17^ These approaches rely heavily on sequence-dependent solubility changes, which can complicate purification after each elongation step and introduce additional processing burdens that are challenging to automate and scale. Although membrane-based LPPS can also encounter solubility limitations as peptide chain length increases, it can reduce the operational complexity associated with repeated precipitation or liquid–liquid extraction workflows.

Organic solvent nanofiltration (OSN) provides an alternative strategy for separating support-bound peptide intermediates from reagents and byproducts in LPPS using solvent-stable membranes, often in the ∼1–2 kDa molecular weight cutoff (MWCO) range (sometimes considered between nanofiltration and ultrafiltration), to retain high molecular weight species while allowing smaller molecules to permeate, thereby streamlining purification. So et al. reported using a soluble anchor attached to a growing 5-mer peptide, where a ceramic membrane was used to separate reaction debris.^18,19^ Yeo at al improved on this by attaching multiple growing peptides to a central hub molecule, increasing the molecular weight difference between the peptide complex and reaction debris to facilitate separation by polymeric membranes, in the PEPSTAR (liquid-phase peptide synthesis by nanostar sieving) process. Peptides up to 10 amino acids were synthesized with superior purity compared to those produced by solid phase peptide synthesis (SPPS). However, in liquid-phase processes, high peptide concentration is desirable to minimize solvent volumes for purification. Yeo et al. reported that precipitation occurred at concentrations above 20 mM, limiting process efficiency.^20^

A key limitation of LPPS is the decline in coupling efficiency as peptide chain length increases, which makes long-peptide preparation challenging.^21–23^ Fragment assembly, the convergent coupling of presynthesized peptide fragments, addresses this problem by reducing the number of elongation steps required and enabling more efficient construction of long sequences.^24^ Fragment condensation in LPPS has therefore re-emerged as a powerful strategy to assemble longer peptides from protected fragments while retaining the solution-phase advantages of homogeneous reaction control and the potential for scale-up.

Recent innovations in tag-assisted and hydrophobic-support strategies (hydrophobic benzyl-alcohol tags, safety-catch linkers, and semipermanent C-terminal protecting groups) have further expanded the utility of fragment condensation. These approaches improve fragment solubility and enable purification by precipitation or phase separation.^25^ As a result, increasingly complex sequences have become accessible: for example: h-Ghrelin (28 residues) was synthesized in 68% yield, and Bivalirudin (20 residues) in 85%.^26^

Liu and co-workers extended hydrophobic-support-assisted LPPS to the therapeutic peptide semaglutide (31 residues), assembling six fragments, and reported an overall yield of 28%, after precipitation-based recovery followed by chromatographic purification.^27^ Industrial and scale-oriented implementations of fragment condensation have also been reported. For example, process routes for the HIV fusion inhibitors enfuvirtide (36 residues) and related 39-mer sequences split peptides into multiple SPPS-prepared fragments that were assembled in solution; intermediates were isolated by precipitation or extraction, yielding peptides of ∼60–70% purity and <30% overall yield. Final polishing by RP-HPLC was required, underscoring the solvent intensity and limited scalability of these workflows.^28^

Recently, Frederick et al. demonstrated the synthesis of Tirzepatide, a 42-mer peptide synthesized via fragment condensation at 15–20 mM peptide concentration using OSN without intermediate chromatographic purification.^29^ Unlike soluble-support LPPS approaches, Lilly’s process did not employ a soluble support, and because the fragment molecular weights were similar to that of the growing peptide, excess peptide fragments were not removed between coupling steps. While this simplified process operations, it also allowed excess fragments to persist and participate in undesired side reactions during subsequent couplings, which in turn required larger fragment excesses in later stages. The final 42-mer peptide was obtained in 46% crude yield and >70% crude purity, but chromatographic purification was still required to remove byproducts, resulting in a solvent-intensive bottleneck for manufacturing.^30^

More recently, Jalan et al. reported a gram-scale convergent synthesis of Tirzepatide via a two-fragment Native Chemical Ligation (NCL) strategy employing a 20-mer peptide thioester and a 19-mer N-terminal cysteinyl fragment. Their tangential flow filtration (TFF)-assisted NCL/desulfurization process enabled high crude ligation purity (∼89%) and good intermediate purification prior to desulfurization, ultimately affording Tirzepatide in ∼45% overall yield with 93% final purity following RP-LC purification. While this approach represents a major advance in convergent peptide manufacturing, it still depends on specialized NCL chemistry and final chromatographic purification.^31^

Similarly, Han et al. recently reported the convergent total synthesis of Tirzepatide via a two-fragment α-ketoacid–hydroxylamine (KAHA) ligation strategy using a 15-mer α-ketoacid segment and a 23-mer cyclic hydroxylamine segment. This method enabled native residue-forming ligation at a challenging Lys–Ile junction under mild AcOH conditions, affording purified Tirzepatide in 42% isolated yield after HPLC purification. Although highly innovative from a ligation chemistry perspective, the process relies on custom hydroxylamine building blocks and preparative chromatography.^32^

In this study, we sought to eliminate the need for chromatography through innovation to the PEPSTAR platform. By anchoring the growing peptide to a bulky 8 arm, 20 kDa nanostar support, comprising solubilizing polymers between the hub and each linker on the support, we aimed to differentiate the growing peptides from excess fragments, enabling their efficient separation. The use of ∼2.5 kDa polyethylene glycol (PEG) on each arm solubilizing polymers was deployed to enhance solubility and seek to increase peptide concentrations from 20 mM to 40 mM during coupling.

Accurate monitoring of peptide synthesis is desirable to ensure efficient coupling reactions and high final yields, and methods such as chromatography and mass spectrometry have been widely used for this purpose. However, the high molecular weight 8 arm 20 kDa polydisperse supports are not amenable to direct injection on UHPLC-MS, and the interpretation of masses is complicated by the polydispersity of the PEG solubilizing polymers. Global deprotection of samples withdrawn from the synthesis at each step is laborious, and the conditions for deprotection themselves can generate impurities that further complicate monitoring of the progress of each coupling cycle.

Photolabile linkers have gained significant attention in recent research due to their ability to release target molecules through simple irradiation. The process of photochemical release can be achieved without the need for additional reagents, often under mild reaction conditions.^33^ Nitrobenzyl-based linkers are among the most commonly used linkers in solid-phase peptide synthesis to release the peptide from the solid support.^34^ this study, we have integrated photolabile linkers into the nanostar support to facilitate real-time monitoring of reaction progress, while preserving the acid-labile linker for final cleavage of the peptide from the soluble support.

Using the solubilizing polymer nanostar and photolabile linker, we synthesized a 39-mer peptide via the assembly of eight presynthesized peptide fragments. The peptide fragments were synthesized via SPPS with supplier-reported UPLC-UV purities greater than 97%. Finally, the 39-mer peptide was cleaved from the nanostar support and isolated via OSN, eliminating the need for chromatography.

SPPS remains the dominant and widely adopted method for peptide production, providing a robust and versatile platform for both laboratory and industrial applications. In parallel, there is growing interest in exploring complementary strategies that may expand the toolkit for peptide synthesis. In this study, we set out to improve LPPS through fragment assembly as a complementary, future-oriented approach. Short fragments were first prepared by SPPS and then coupled in solution, reducing the number of elongation steps needed for long sequences. Through this hybrid design, we aimed to develop a chromatography-free fragment assembly platform based on the PEPSTAR nanostar support, offering a complementary path alongside SPPS for efficient and scalable peptide synthesis.

## Results and Discussion

### The PEPSTAR Synthesizer

The core operational principles of the PEPSTAR synthesizer used in this work are illustrated in Figure 1a. The nanostar support is dissolved in the desired solvent and introduced into the reaction loop vessel. The fluid circulation carries the dissolved nanostar support through the membrane cell before returning to the reaction loop vessel. Reactions are carried out by introducing reagents through the injection outlet in the reaction loop vessel, as the reaction mixture circulates between the reaction loop vessel and membrane cell. This circulation ensures that the reaction occurs throughout the entire system.

**Figure 1:**
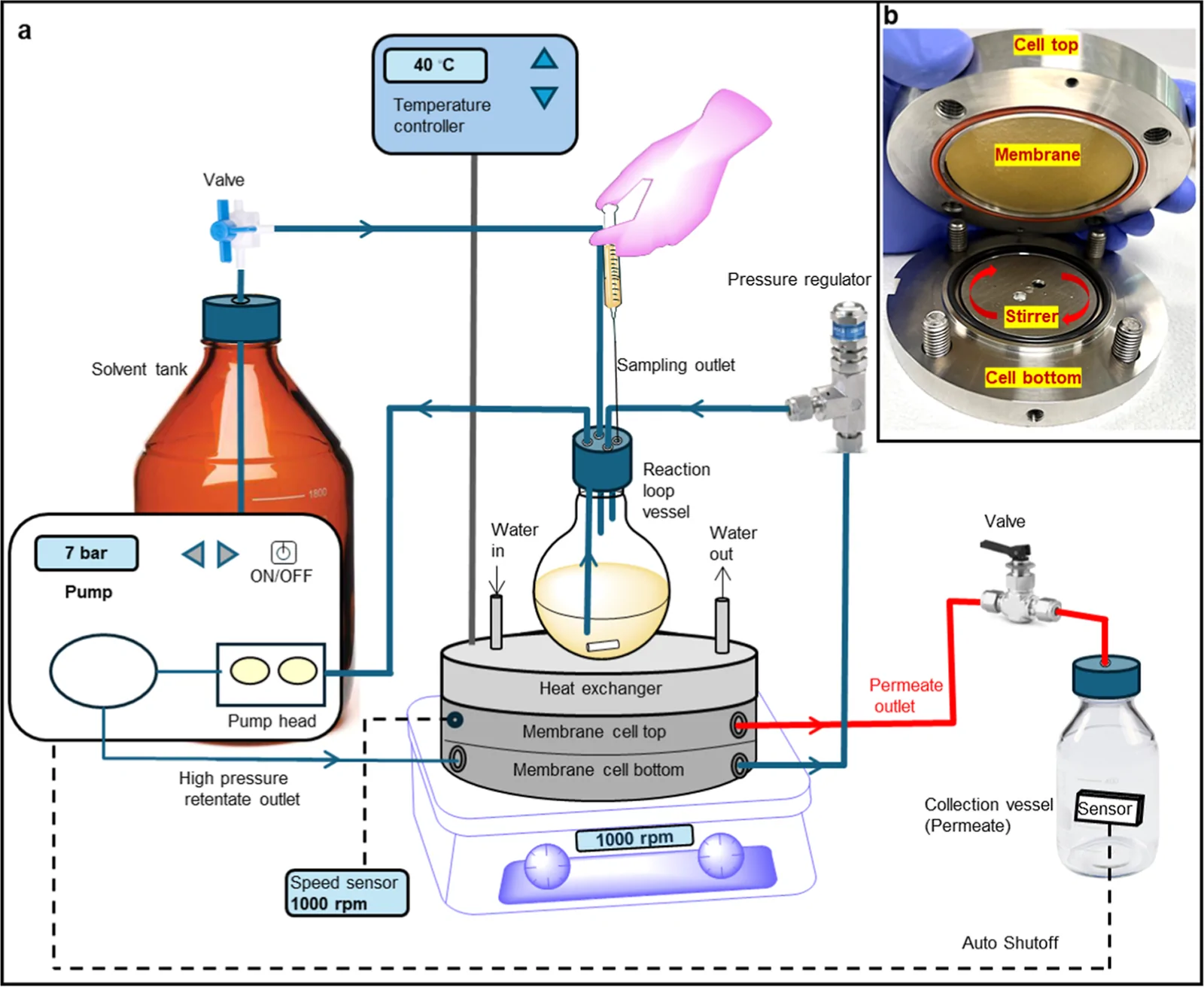
(a) The PEPSTAR synthesizer. The flow-through reactor/membrane separator cell has a working volume of ∼40 mL and membrane area of ∼33 cm^2^. Membrane Area/Volume ∼1.32 cm^2^/mL. (b) Inside view of the membrane cell.

The built-in stirrer ensures effective mixing (Figure 1b), reducing concentration polarization above the membrane. Fluid circulation and pressure are controlled by a dual-piston AZURA pump equipped with a pressure sensor and a stainless-steel pump head, capable of reaching flow rates up to 50 mL/min. An integrated refractive index sensor automatically shuts off the pump when the permeated solvent reaches a predefined volume in the used solvent vessel.

The nanofiltration process achieves selective retention of support-bound growing peptide. By increasing the pressure (7–10 bar) on the retentate side of the membrane during the diafiltration stages, a controlled volume of solvent is forced through the membrane into a collection vessel, while the remaining solvent recirculates to maintain system equilibrium. Small molecules, including unreacted reagents and byproducts, are efficiently removed as they permeate through the membrane, whereas the nanostar support and bound species are retained due to size exclusion. The continuous replenishment of solvent from the solvent tank maintains a constant volume, preventing dilution effects and ensuring optimal reaction conditions.

### Selection of Soluble Support (Nanostar) and Membranes

Efficient nanostar sieving relies on a membrane with high degree of selectivity for the nanostar support, ensuring high rejection of the support while allowing unwanted species to pass through. To improve selectivity, we evaluated commercially available branched PEG-amine stars **1**, **2**, and **3** (Figure 2). In optimized runs using Nanostar 3, peptide synthesis was performed at a fixed 40 mM concentration throughout all fragment coupling cycles in 60:40 DMF/MeCN (v/v), while diafiltration was typically conducted at concentrations up to 55 mM without solubility issues.

**Figure 2:**
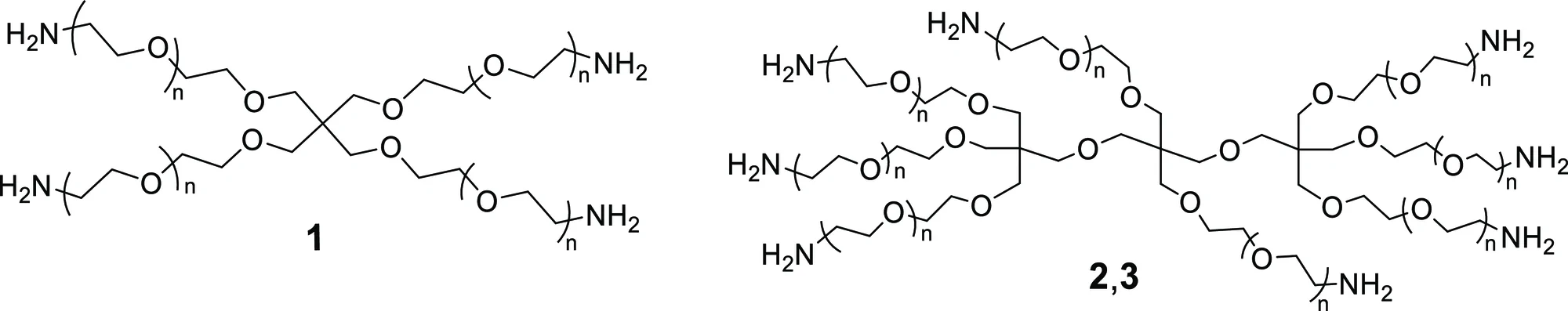
Nonuniform mass nanostars (4-arm 10 kDa PEG **1**, 8-arm 10 kDa PEGs **2** and 8-arm 20 kDa PEGs **3**).

Based on our previous studies^35,36^ we selected a 17 wt % polybenzimidazole membrane (PBI) cross-linked for stability,^35^ and with grafted oligomers on the surface to mediate selectivity and reduce fouling.^36^ Additionally, a 12 wt % polyetheretherketone membrane (PEEK) was also used to initiate peptide synthesis.^37^ The PEG nanostars **1** and **2** were highly rejected by the PEEK membrane, with rejections exceeding 99.5% after the installation of the first linker, and rejection increased further as peptide fragments were added.

Although the PEEK membrane exhibited relatively higher rejection for the 8-arm 10 kDa nanostar **2** compared to the PBI membrane, which was 98.5%, additional diavolumes (the cumulative volume of wash solvent introduced to the operation at a given time in respect to the synthesizer volume) were required to completely remove peptide fragments because the rejection of fragments was higher with the PEEK membrane (55%) than with the PBI membrane (35%). This increased washing requirement negatively impacted the overall yield of the final peptide **9**.

Furthermore, the PEEK membrane exhibited signs of tightening after several cycles when the pressure set between 10 and 20 bar, likely due to compaction under sustained higher pressure, which increased fragment rejection and reduced process efficiency. As a result, further optimization led to the selection of the 8-arm 20 kDa PEG nanostar **3** in combination with the 17% PBI membrane. The PBI membrane proved to be more stable and pressure-resistant than the PEEK membrane. Additionally, the PBI membrane facilitated more efficient fragment removal, requiring fewer diafiltration volumes due to its lower rejection of fragments under identical solvent conditions 60:40 DMF/MeCN (v/v).

The use of PEG nanostar **3** significantly improved nanostar retention, achieving 99.9% rejection on PBI, minimizing the loss of PEG-peptide, and ultimately leading to a higher yield. Furthermore, PEG nanostar **3** enhanced the solubility of the growing peptide, enabling the peptide concentration to be doubled from 20 mM to 40 mM without solubility issues.

During peptide syntheses, chromatographic peaks corresponding to peptide-PEG species were observed to broaden, making chromatography an unreliable tool for assessing reaction completion. In previously work with a three-point defined low molecular weight hub,^20^ mass spectrometry was used to confirm successful coupling reactions by detecting the disappearance of masses corresponding to partially reacted nanostar species. However, with the transition from a uniform-mass nanostar to a polydisperse nanostar, this approach was no longer feasible.

To overcome these limitations, a small portion of the reaction mixture was taken, and the peptide was cleaved from the polydisperse nanostar to enable monitoring by mass spectrometry. Initially, cleavage was performed under global deprotection conditions; however, this process required the removal of nonvolatile solvents and redissolving the peptide-bound support in concentrated TFA for up to 3 h, making it impractical for routine monitoring. To address this limitation, a photolabile linker was introduced for reaction analysis and monitoring.

### Incorporating a Photolabile Linker for Efficient Monitoring

We introduced a photolabile linker **4** which enables real-time reaction assessment with minimal sample preparation, improving the efficiency and accuracy of peptide synthesis monitoring. The photolabile linker **4** was synthesized (see Supporting Information) and installed prior to the Rink amide linker **8**, an acid-labile linker typically used in peptide synthesis. The linker **4** is cleaved upon exposure to specific wavelengths of light (365 nm in this study), releasing the protected peptide-intermediate **6** for analysis in minutes without the need to remove the reaction solvents (Figure 3). This feature allows rapid assessment of each coupling step, reducing the risk of incomplete reactions.

**Figure 3:**
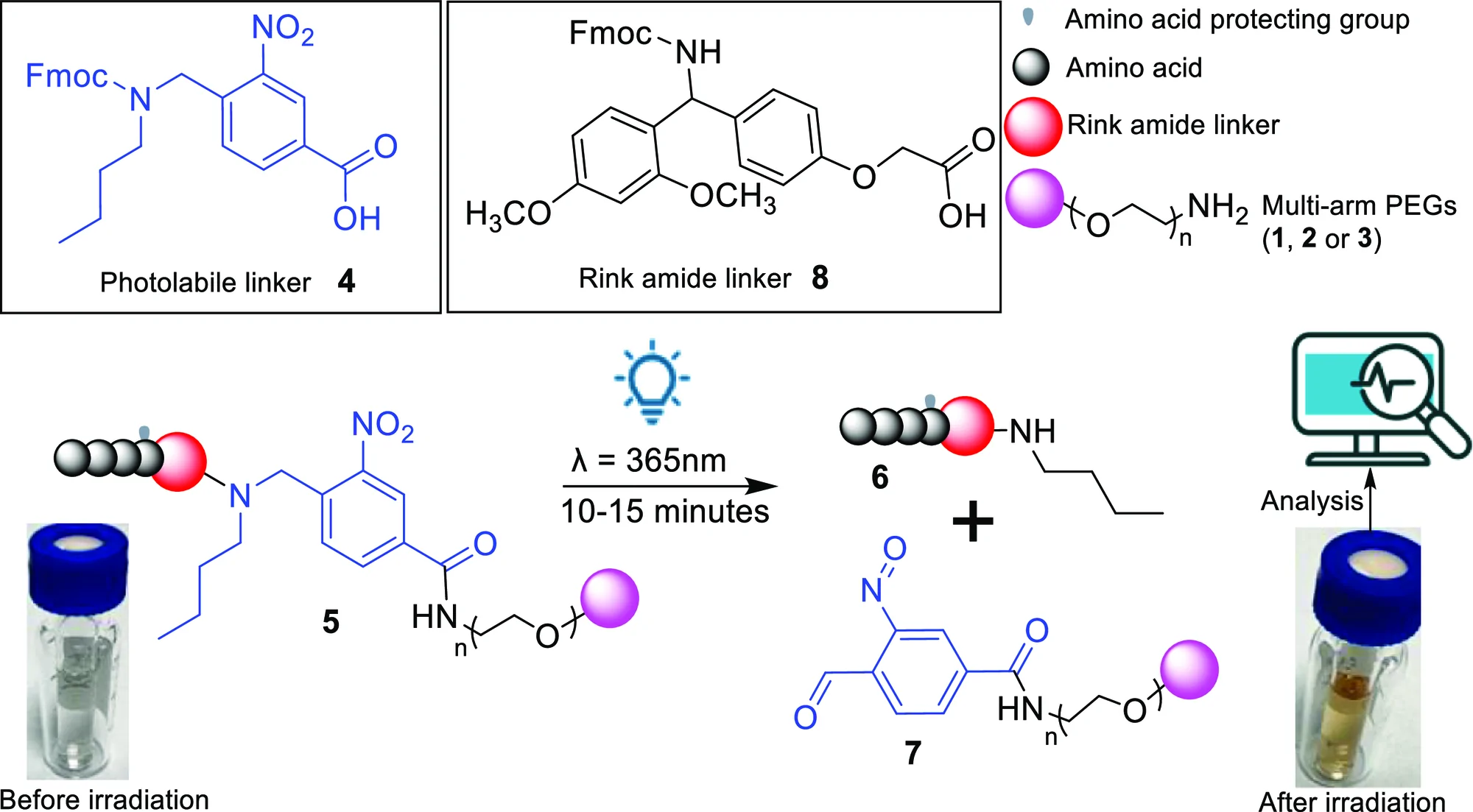
Photocleavage of a peptide from the peptide-bound nanostar **5** to yield a uniform mass peptide species **6** that can be analyzed by UHPLC-MS.

### Synthesis of a 39-Mer Peptide

To demonstrate PEPSTAR’s potential for large-scale peptide synthesis, a 39-mer peptide **9** was selected as a target by collaborators at Eli Lilly and Company (Figure 4a).

**Figure 4:**
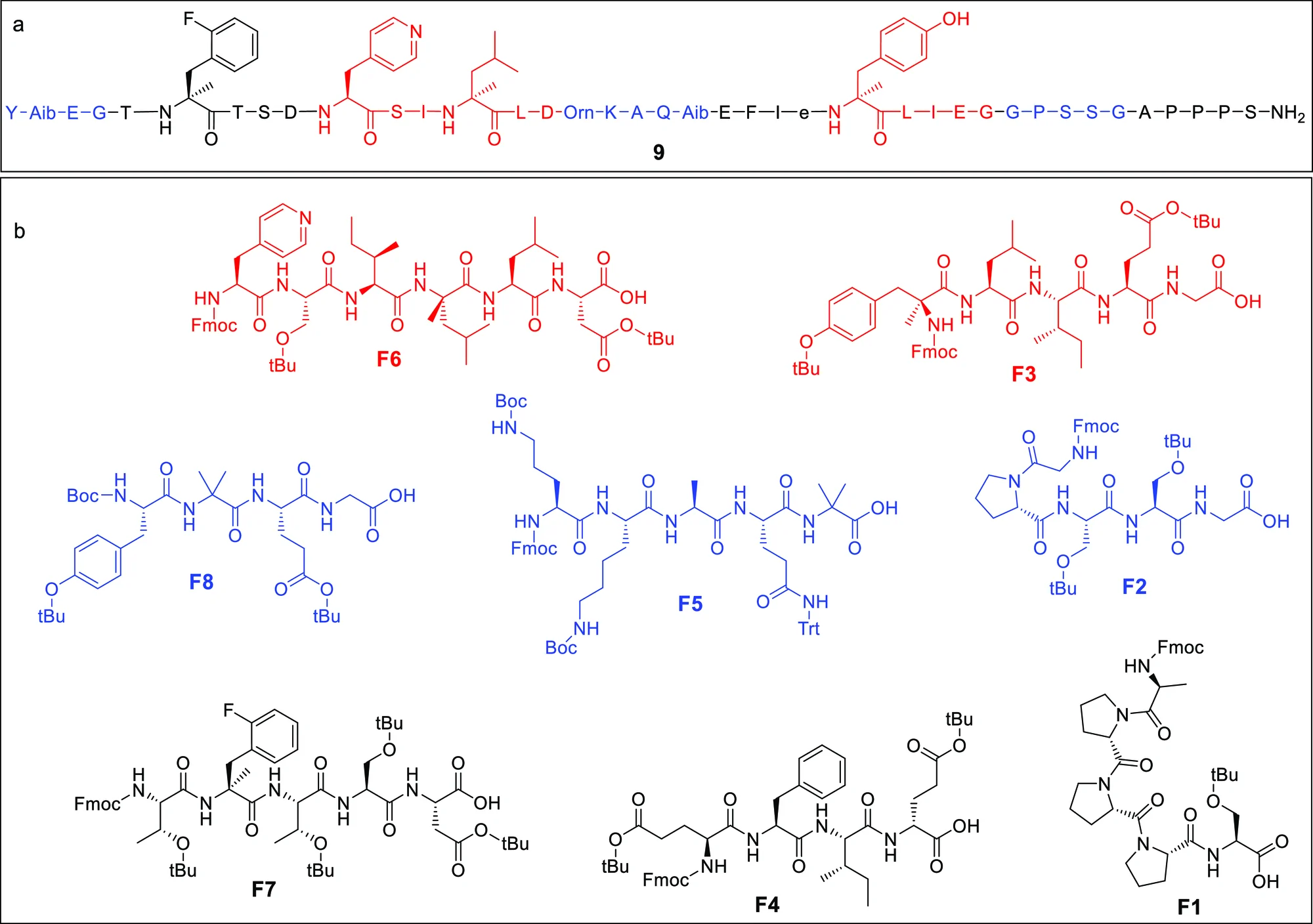
(a) The structure of 39-mer peptide **9**. (b) The structures of the fragments used to synthesize peptide **9**. Unnatural amino acids are given three-letter codes or drawn out fully. A lowercase letter in the sequence denotes a D amino acid.

Linear peptide syntheses suffers from reduced coupling efficiency as the chain length increases, regardless of whether the synthesis occurs in the solid or liquid phase. To address this challenge, we adopted a convergent synthesis strategy, wherein peptide fragments with side-chain protecting groups intact are synthesized and subsequently assembled into the target peptide **9**. This strategy significantly reduces the number of couplings required at longer chain lengths. Our objective was to completely remove excess peptide fragments after each coupling using OSN without significant yield loss. Since larger fragments are more challenging to remove using OSN, fragment size was a crucial consideration in our experimental design (Figure 4b).

The purity of the peptide fragments as synthesized by the supplier are listed in Table 1 and determine the maximum achievable purity of the final assembled peptide. Multiplying the purity values of individual fragments (0.9657 × 0.9765 × 0.9844 × 0.9849 × 0.9733 × 0.9723 × 0.9901 × 0.9753), the highest theoretical purity of the final peptide is estimated to be 83.5%.

**Table 1:** Purity of the Fragments as Provided by the Supplier

fragment	molecular weight	UPLC purity	physical property	color
F1	745.86	96.57%	solid	white
F2	737.84	97.65%	solid	white
F3	942.15	98.44%	solid	white
F4	871.03	98.49%	solid	white
F5	1209.43	97.33%	solid	white
F6	1056.29	97.23%	solid	white
F7	1048.24	99.01%	solid	white
F8	664.79	97.53%	solid	white

The synthesis procedure is outlined in Figure 5. Initially, a photolabile linker **4** was attached to the nanostar **3**. The photolabile linker allowed for facile cleavage of the peptide from the nanostar for monitoring the synthesis. A sample from the synthesizer can be taken, diluted, subjected to light at 365 nm for 10 min, and injected into UHPLC to quickly obtain information about the progress of the synthesis. Subsequently, a Rink amide linker **8** was installed. This acid-labile linker cleaves during global deprotection at the end of the synthesis, yielding the desired peptide with a C-terminal amide. The Rink linker also aids in the analysis of the peptide intermediate when photocleavage is performed, as it remains bound to the peptide and increases its UV response during UHPLC analysis. The fragments were then added sequentially. The coupling cycle for both linkers and each fragment was identical. Each linker or fragment was preactivated separately before being added to the support or support-bound intermediate. Following the coupling of the linker or fragment, piperidine was used to cleave the Fmoc group, and diafiltration (nanostar sieving) was employed to remove reagents and byproducts while retaining the support-bound intermediates. After the final fragment was added, global deprotection was performed, and the peptide was precipitated. Finally, the nanostar debris was removed from the peptide using OSN, resulting in the final product.

**Figure 5:**
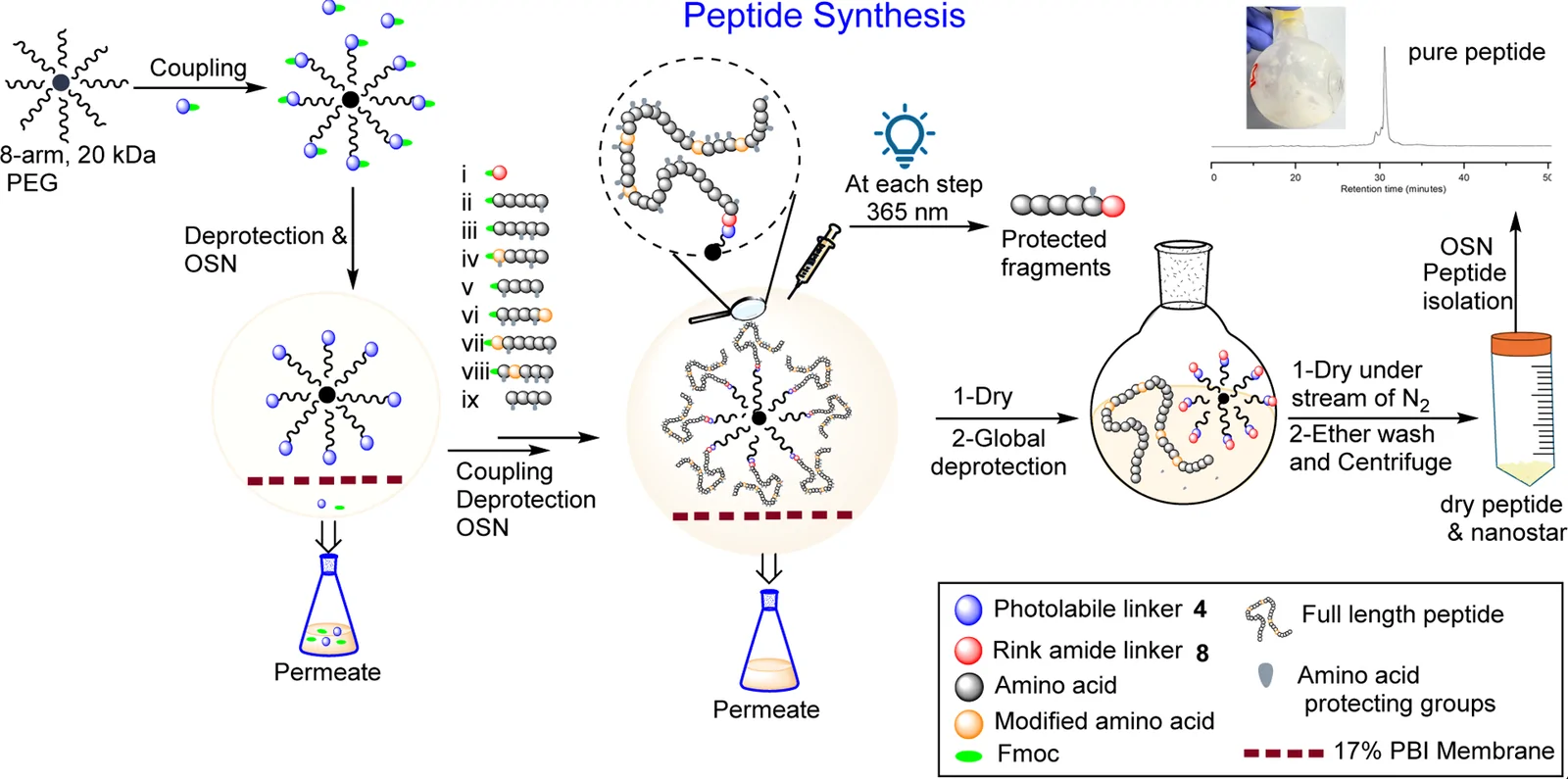
Synthesis strategy of peptide **9**. Conditions: couplings, 1.5 eq. Fragments or linkers/arm, 3 eq. DIC/arm, 3eq HOBt/arm, 60:40 DMF/MeCN, 40 °C, 6 h; Fmoc deprotection: piperidine 5 v/v %, 40 °C, 30 min; Global deprotection: 93:3:3:1 trifluoroacetic acid/water/triisopropylsilane/dithiothreitol, rt, 3 h.

Optimization of the synthesis led to the following method, summarized in Figure 5. We found that solvent mixture of 60:40 DMF/MeCN (v/v) provided improved solubility during the synthesis and was compatible with the 17% PBI membrane, which remained stable. Therefore, this solvent mixture was used throughout the synthesis.

Linkers and Fragments were preactivated in a flask with DIC/HOBt for 15–20 min before being filtered and added to the synthesizer.

Nanostar **3** was highly rejected by the PBI membrane, allowing excess fragments to be easily washed away after each coupling and deprotection step. Additionally, nanostar **3** was easily isolated from the final peptide due to the size difference between nanostar **3** (20 kDa) and the final peptide (4206.08 g/mol).

The support-bound peptide is not recovered from the synthesizer until after the final fragment is attached. This feature makes PEPSTAR highly amenable to automation, as the synthesis is performed by simply injecting reagents into the synthesizer’s injection outlet and adjusting the pressure regulator to initiate diafiltration when desired. Sensors for the automatic shut-off of the pump supplying pressure and circulation allow the operator to leave diafiltrations unattended for hours without the risk of unnecessary diafiltration, which could result in decreased yield and wasted solvent. This was highly convenient as diafiltrations (nanostar sieving) took between 3 and 12 h, increasing in duration as the synthesis progressed. This was due to decreasing membrane permeances (0.0567–0.0076 LMH/bar) which we attributed to the reaction mixture becoming more viscous as consecutive fragments were attached to the nanostar.

The attachment of the photolabile linker was initiated by injecting the previously activated linker into the synthesizer containing the solution of nanostar **3** through the sampling outlet. The coupling was confirmed to have gone to completion by UHPLC analysis, after which piperidine was added to cleave the Fmoc, generating a reaction mixture that was 5% piperidine by volume. Similarly to previous work, no separation step was needed to remove excess linker/fragment prior to the addition of piperidine, as the piperidine was found to instantaneously quench the activated linker/fragment before Fmoc deprotection, ensuring no double coupling took place. After 30 min, the deprotection was completed (confirmed by UHPLC), and excess coupling and deprotection reagents, along with N-fluorenylmethylpiperidine (DBF-pip), were removed by constant volume diafiltration. Piperidine (5% v/v of the synthesizer volume at the time of addition) was found to be suitable for the removal of Fmoc, with diafiltration started 30 min after the addition of piperidine. The volume in the synthesizer increased as reagents are added and was then reduced by performing diafiltration, allowing air to be drawn in instead of using replacement solvent to concentrate the reaction solution. The target volume for the synthesizer was roughly 35 mL. Once this volume was reached, the synthesizer was closed to air causing solvent to be drawn instead of air, keeping the volume constant during the rest of the diafiltration. Using a 17 wt % PBI membrane, 10 diavolumes of solvent was found to completely remove the excess fragment, as determined by UHPLC-MS with an ELSD detector. This is a significant advantage of nanostar sieving versus other OSN based fragment assembly methods, which are unable to isolate the growing peptide from the excess fragments used in coupling.

Removing piperidine before the next coupling cycle is vital, as piperidine will quench the next monomer or, in some cases, deprotect it, resulting in unwanted double coupling. An upper limit of 0.01% v/v piperidine was chosen, and it was found that 8–10 diavolumes (280–350 mL) were required to reduce the concentration of piperidine to this level, as determined by gas chromatography. In the case of the photolabile and Rink linkers, this amount of solvent was sufficient to remove the excess linkers to below quantifiable levels, as determined by UHPLC analysis.

When reporting the number of equivalents of reagents, we report the number of equivalents per arm of the nanostar rather than per equivalent of the nanostar itself to ensure our methods are easily comparable with other protocols. We followed the same procedure for the coupling of the Rink linker and the fragments: 1.5 equiv of Rink/fragment were preactivated with 3 equiv of HOBt and 3 equiv of DIC. Diisoprobyl urea (DIU) was filtered out before injecting the activated Rink/fragment into the synthesizer via the sampling outlet.

At any stage, samples can be collected from the synthesizer for analysis, and photocleavage can be performed to gain additional insights into reaction progress beyond what is possible through direct analysis of the reaction mixture. When analyzing coupling reactions after photolysis, we specifically looked for the cleaved protected product (e.g. **11**) in the 230 nm spectrum, and any unreacted peptide, if present. The presence of an unreacted peptide intermediate in the analysis indicates that the reaction has not yet reached completion.

For example, we collected a sample from the synthesizer, diluted it with acetonitrile, and submitted it for UHPLC analysis. The sample was then irradiated with 365 nm light for 15 min and resubmitted for analysis. Figure 6 illustrates the photolysis results following the coupling of fragment F1. The UV spectrum initially showed only the nanostar peak at a retention time of 5.2 min before photolysis. However, after 15 min of photolysis, we observed a physical change in the sample color from clear to brown. Additionally, a new peak corresponding to the cleaved product **11** appeared at a different retention time of approximately 4.0 min in the UV spectrum. The exact mass of the cleaved product was confirmed using mass spectrometry.

**Figure 6:**
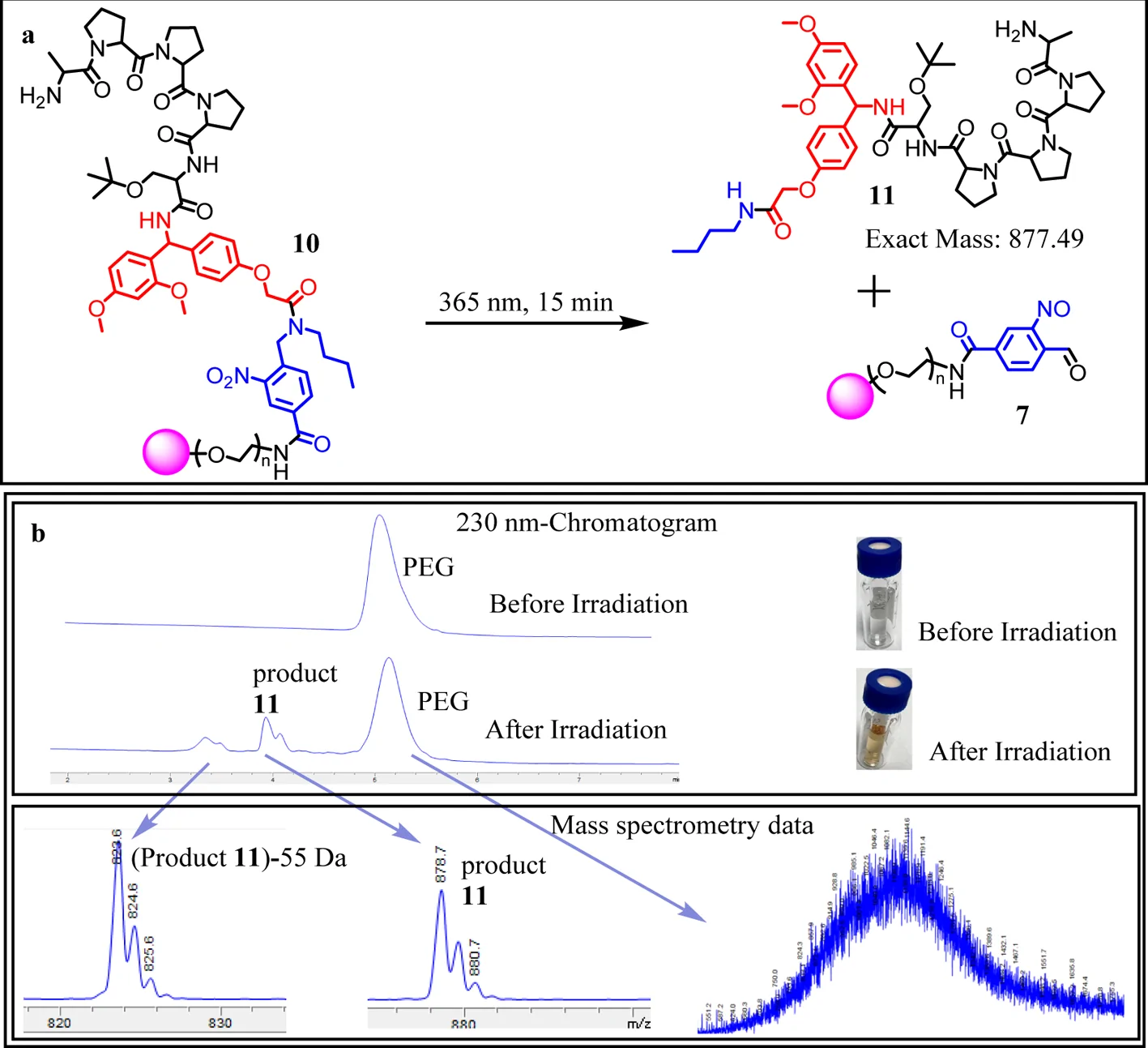
(a) Photolysis scheme of **10**. (b) UHPLC-MS data for the cleaved intermediate **11**.

Moreover, the spectrum displayed an additional peak at a retention time of 3.4 min, which had the same exact mass as cleaved product **11**, minus 55 Da. We hypothesize that this 55 Da difference may be attributed to the hydrolysis of the butyl group from the photolabile linker during photolysis or UHPLC analysis. The appearance of two peaks in the UV spectrum of Product 11 is attributed to the presence of a chiral center in the Rink amide linker, which results in the formation of photolysis products as both R and S isomers. Notably, no peaks corresponding to unreacted species were detected on the UV chromatogram, nor was any unreacted mass observed in the mass spectrum, indicating that the reaction had proceeded to completion (Figure 6b).

Following the coupling of fragment **5**, the photocleaved peptide was observed to coelute with the nonuniform mass nanostar, complicating the determination of the coupling efficiency. To assess the reaction outcome, mass spectrometry was used to identify the product and any unreacted species. Extending the coupling reaction to 6 h or longer resulted in the near-exclusive formation of the target peptide sequence, with detectable but insignificant amounts of impurities.

The terminal fragment was designed with a Boc-protected N-terminus, eliminating the need for an Fmoc deprotection step after coupling. To remove residual nonvolatile DMF, along with other reagents and byproducts, the final nanostar-bound peptide was washed with ten diavolumes of a 50:50 methanol/acetonitrile (MeOH/MeCN) mixture. The contents of the synthesizer were then drained into a 500 mL flask, and an additional wash with 50:50 MeOH/MeCN was performed to ensure complete collection of the support-bound peptide.

The solvent was removed under vacuum, and the dry peptide-nanostar was redissolved in a global deprotection cocktail consisting of 93:3:3:1 trifluoroacetic acid/water/triisopropylsilane/dithiothreitol by volume (assuming solid dithiothreitol has a density of 1 g/mL). The support-bound peptide was dissolved in the cleavage cocktail and stirred for 3 h. Afterward, the volatiles were evaporated, and the oily residues were added dropwise into a 50 mL centrifuge tube containing cold methyl *tert*-butyl ether (MTBE). The precipitates were isolated, washed twice with MTBE, and collected by centrifugation to afford the crude mixture of deprotected peptide **9** and nanostar residues.

The UHPLC chromatogram in Figure 7 demonstrated that the precipitated peptide was isolated as a mixture with the nanostar residues. Since it was not possible to isolate peptide **9** from the nanostar using traditional methods such as extraction or precipitation, we decided to use the OSN to isolate the peptide based on size exclusion.

**Figure 7:**
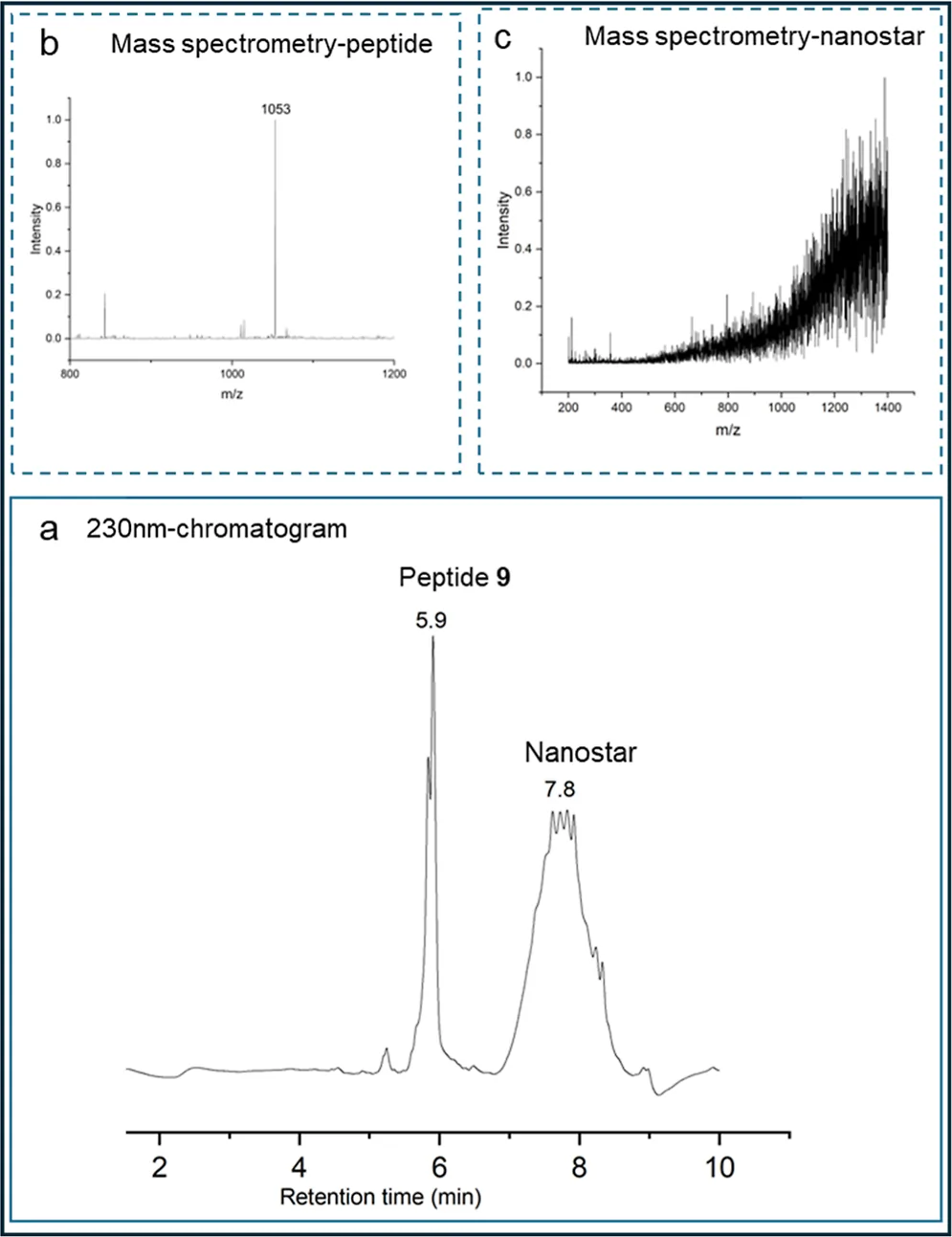
UHPLC chromatogram (230 nm UV absorbance) and mass spectrometry analyses of the support-bound peptide mixture obtained after global deprotection and ether washing. (a) UHPLC chromatogram recorded after global deprotection, showing the peptide 9 peak at a retention time of 5.9 min and the Nanostar residue peak at 7.8 min. (b) Mass spectrum of peptide 9, showing the exact mass of the quadruply protonated ion, (M + 4H)^4+^. (c) Mass spectrum of the Nanostar residue, showing its polydisperse mass distribution.

### Isolation of the 39-Mer Peptide

The separation of the peptide from the nanostar proved challenging. Conventional methods such as extraction and precipitation were ineffective; therefore, nanostar sieving was explored. The initial strategy involved permeating the peptide through a 17% PBI membrane while retaining the nanostar, followed by evaporation of the permeate to recover the peptide. However, the deprotected peptide exhibited a high rejection rate (92%) on the 17% PBI membrane, necessitating large solvent volumes and a time-intensive process.

Attempts to improve separation efficiency using more open PBI membranes (16%, 14%, and 12%) were unsuccessful, as they did not achieve adequate selectivity. Likewise, testing with PEEK membranes showed that tighter variants rejected both the peptide and the nanostar, whereas more open versions permitted both species to pass through. In both cases, precise control over the separation performance was not achievable. Moreover, membrane performance in this solvent system depended not only on nominal MWCO but also on factors such as solvent-induced swelling, peptide conformation, and electrostatic interactions, particularly during aqueous isolation steps. These limitations led to the exploration of commercial membrane alternatives. We speculated that, during the peptide isolation step (i.e., upon detachment of the peptide from the nanostar), separation was governed mainly by electrostatic interactions and peptide conformation. This hypothesis was further supported by our subsequent observations with the PES membrane.

A poly­(ether sulfone) (PES) membrane with a 10 kDa cutoff was selected for further trials. However, this membrane proved unstable in acetonitrile, leading to polymer leaching and subsequent nanostar permeation. Structural visual examination revealed membrane degradation, with visible cracks after exposure to the solvent environment.

Further testing in aqueous-mixtures revealed that the PES membrane exhibited greater stability with higher water content. It remained intact in a 60:40 H_2_O/MeOH (v/v) mixture but became increasingly unstable as the methanol content exceeded 70%, and it degraded significantly in pure methanol or isopropanol. Consequently, a 60:40 H_2_O/MeOH mixture was chosen as the optimal solvent system, providing the minimum methanol concentration necessary to dissolve the peptide-nanostar mixture while maintaining membrane integrity.

Since the deprotected peptide required a more delicate isolation process, a heat exchanger was implemented to maintain the isolation temperature between 16–22 °C, mitigating potential degradation. Initial trials with the 10 kDa PES membrane resulted in peptide rejection rates ranging from 85% to 94%, while nanostar rejection remained constant at 100%. Switching to a 30 kDa PES membrane yielded similar rejection patterns, indicating that molecular weight alone did not account for the high peptide retention.

To investigate potential electrostatic interactions, the effect of pH on membrane performance was examined. Modifying the pH with 0.1–1% ammonium hydroxide did not alter peptide rejection. However, an acidic pH significantly reduced peptide retention. The introduction of 0.1% trifluoroacetic acid (TFA) lowered peptide rejection, and at 1% TFA, complete peptide permeation (0% rejection) was achieved, while nanostar rejection remained at 100%. Since the PES membrane is negatively charged, it was hypothesized that peptide rejection was influenced by charge repulsion. It is likely that at this concentration, the acidic environment neutralized the charges of both the PES membrane and the peptide, reducing electrostatic repulsion and facilitating efficient peptide isolation. This optimization reduced solvent consumption and isolation time to approximately three diafiltration volumes instead of 20–30 diafiltration volumes with the peptide in neutral solvent solution.

## Solvent Exchange and Final Peptide Isolation and Future Directions

Following successful peptide isolation, solvent exchange was necessary to transition from the 59:40:1 H_2_O/MeOH/TFA (v/v/v) mixture to an organic solvent such as methanol, enabling efficient evaporation without excessive heating. To achieve this, the peptide was retained using a tighter nanofiltration membrane. A 1 kDa poly­(piperazine-amide) (PPA) membrane, stable in both pure methanol and the 59:40:1 H_2_O/MeOH/TFA (v/v/v) mixture, was employed.

The peptide solution was first concentrated to 35 mL via filtration, after which methanol was gradually introduced to replace water and TFA. After eight diafiltration volumes, the solvent was evaporated, yielding the peptide as a white powder. While this approach successfully retained the peptide with 100% rejection while allowing complete permeation of water and TFA, prolonged exposure to the MeOH in acidic solution led to the formation of impurities not initially present in the crude mixture.

UHPLC-MS analysis (Figure 8) identified these impurities as methyl esters, formed via esterification of certain amino acids in the peptide in the presence of methanol and acid. This side reaction was not observed during PES membrane isolation but occurred during the extended diafiltration process with the PPA membrane. To mitigate esterification, an alternative workflow was devised (Figure 9). Methanol was replaced with isopropanol (IPA) while maintaining the same procedure. Although peptide rejection on the PES membrane increased to 25%, complete isolation was achieved after five diafiltration volumes. No esterification was detected under these conditions. To further suppress the esterification reaction during subsequent solvent exchange, 2% v/v triethylamine was added dropwise to the solution of peptide in 59:40:1 H_2_O/IPA/TFA (v/v/v) mixture before transitioning to methanol on the PPA membrane. This modification allowed efficient solvent exchange while preventing esterification. The peptide **9** was ultimately collected and analyzed, affording a white powder with 88% yield, without requiring any chromatographic purification.

**Figure 8:**
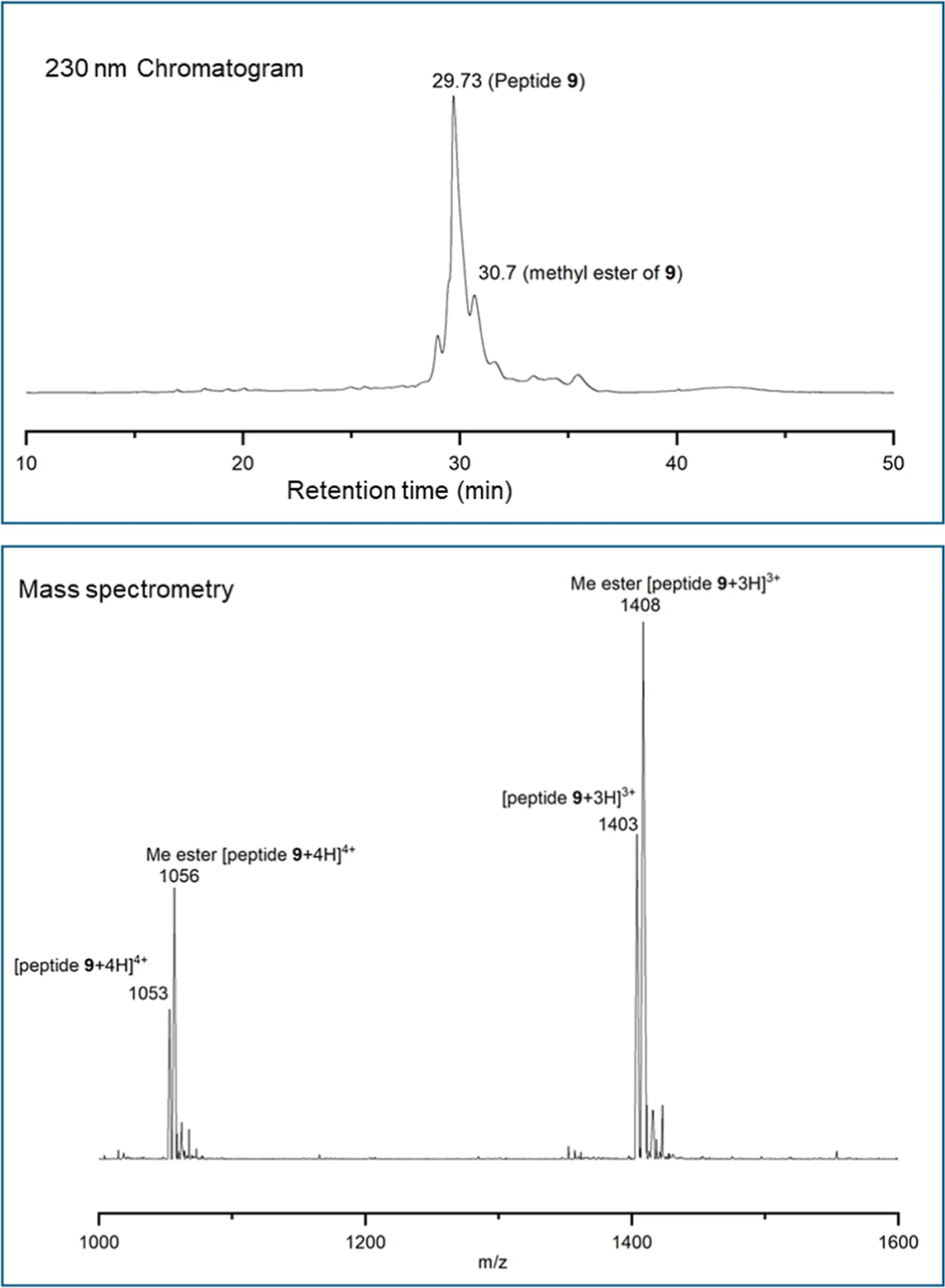
UHPLC chromatogram (UV absorbances and corresponding mass spectrometry) of the peptide methyl ester.

**Figure 9:**
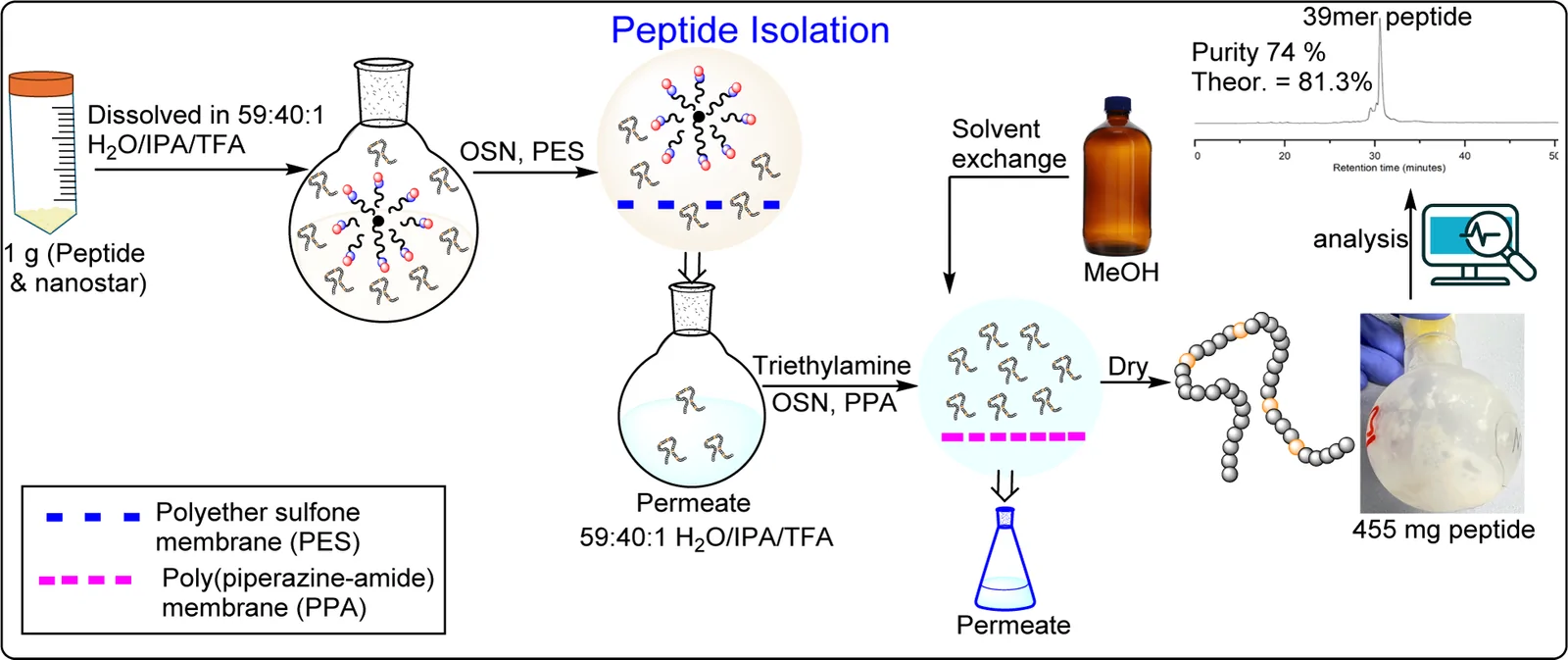
Isolation strategy of the synthesized peptide **9**.

The collected peptide had a purity of 74% (Figure 10), which is not far from its maximum achievable purity of 83.5%. The 9.5% impurity consists primarily of tyrosine loss, epimerization, peptide **9** minus **F8** ≤ 1%, and peptide **9** minus **F7** ≤ 1%. These results from a nonoptimized process highlight the promise of the PEPSTAR methodology in peptide synthesis and purification, demonstrating its ability to achieve high purity while minimizing unwanted byproducts. With appropriate process modifications, this approach has the potential to enable chromatography-free peptide synthesis.

**Figure 10:**
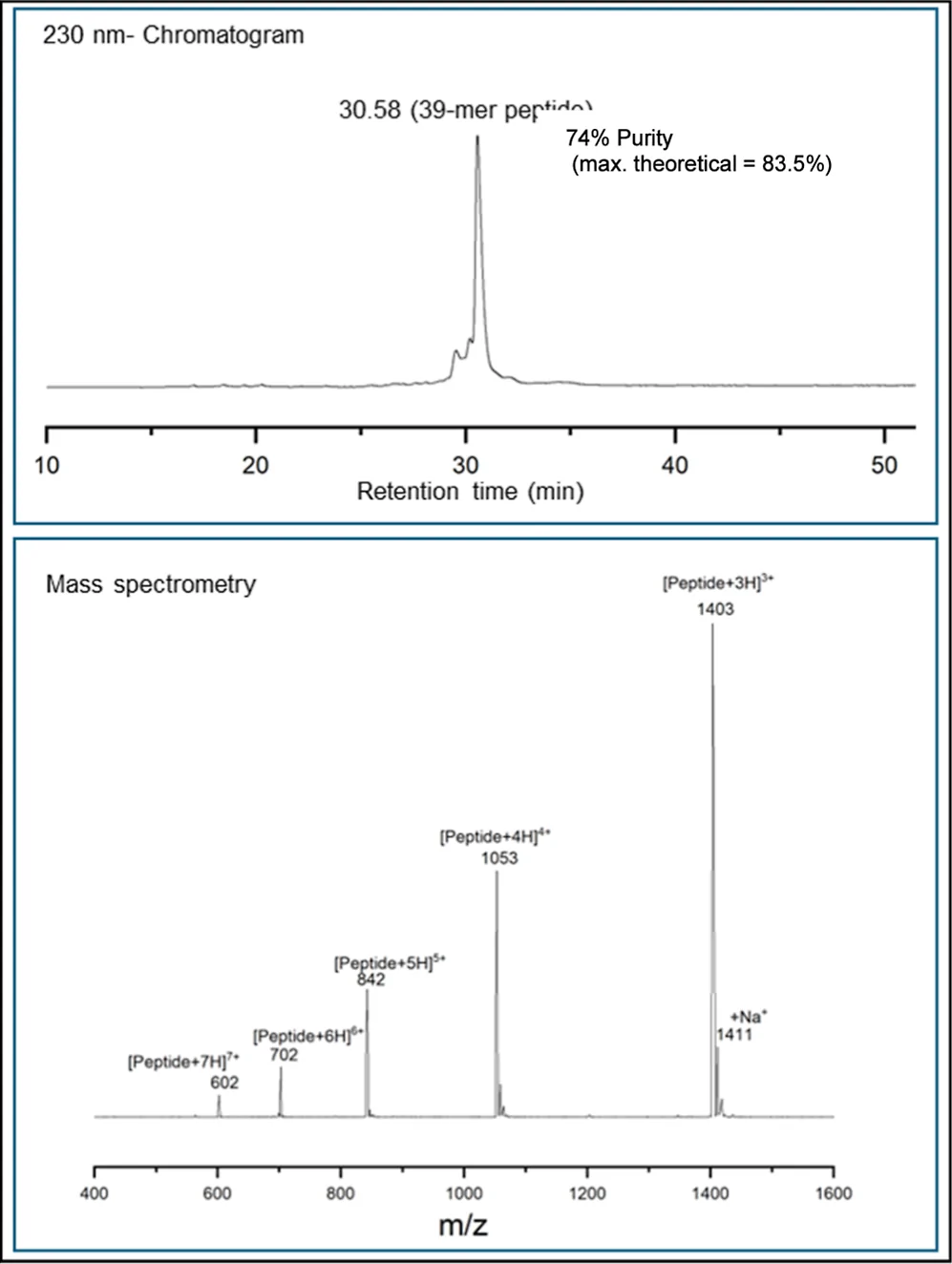
UHPLC chromatogram (UV absorbances and corresponding mass spectrometry) of the isolated 39-mer peptide **9**.

The peptide synthesis was performed five times, as outlined in Table 2. In the first two runs, a 4-arm 10 kDa nanostar (Nanostar **1**) was used. However, due to its 99.5% rejection rate, the maximum achievable yield was low, and effective separation of the peptide from the PEG was not possible, with the result that we were not able to calculate the yield and the purity for the first two runs. A bulkier 8-arm 10 kDa nanostar (Nanostar **2**) was employed. While this nanostar exhibited good rejection on PEEK membrane, it required larger diafiltration volumes to remove the excess fragments at each step, negatively impacting the final yield (Run 3). After peptide cleavage from run 3 a 10 kDa cutoff PES membrane was also tested, which allowed peptide separation from PEG but resulted in a 70% peptide rejection rate, necessitating longer processing times and increased solvent consumption.

**Table 2:** Summary of Peptide Purity and Yield Obtained in Different Synthesis Runs

run	nanostar	peptide conc. mM	purity %	yield %	membrane (synthesis)	membrane (isolation)
1	4arm-10K, **1**	20	N.A	N.A	PBI 17%	N.A
2	4arm-10K, **1**	20	N.A	N.A	PBI 17%	N.A
3	8arm-10K, **2**	30	64	62	PEEK	PES (10 kDa) + PPA
4	8arm-20K, **3**	20	70	67^a^	PBI 17%	PES (30 kDa) + PPA
5	8arm-20K, **3**	40	74	88	PBI 17%	PES (30 kDa) + PPA
6	8arm-20K, **3**	40	75	87	PBI 17%	PES (30 kDa) + PPA

a The yield is lower than in run 5 and 6 due to a pump leak that occurred during run 4.

Subsequently, we improved the performance by using Nanostar **3**, an 8-arm 20 kDa PEG. The synthesis was conducted three times. In the first attempt, run 4 (20 mM conc.) a pump leak occurred due to seal damage, leading to a lower yield compared to the p attempt. Run 5 and 6 performed with a 40 mM peptide concentration (168 g/L), double the concentration of the previous runs (20 mM, 84 g/L), the process proceeded smoothly without solubility issues. However, after coupling with **F5**, the permeate flow rate decreased to 0.4 mL/min (Permeance ≈ 0.0076 LMH/bar), requiring over 12 h to complete the diafiltration. By utilizing an auto shutoff sensor, the process was completed overnight without human intervention.

For this study, lower purity fragments were used, as their syntheses were not optimized. The current fragment purities range from 96.57–99.01%. Future optimizations will focus on incorporating higher-purity fragments >99.5%, which is expected to improve both process yields and final peptide purity.^29^ Additionally, efforts will be directed toward developing a more selective membrane capable of one-step purification. By enabling peptide permeation in a single step using organic solvents such as methanol or a methanol/acetonitrile mixture, this approach would further reduce processing time and solvent consumption, enhancing the efficiency and sustainability of peptide synthesis.

Linear peptide synthesis in the liquid phase has generally been successful for shorter sequences (≤20 amino acids), whereas longer peptides are more commonly obtained through fragment assembly strategies, often using SPPS for fragment synthesis as in Table 3. When compared with the published reports that employed fragment-based LPPS, the present study demonstrates an attractive outcome, particularly in terms of crude yield and purity for peptides of comparable length (Figure 11).

**Table 3:** Comparison of Peptide Synthesis Approaches and Outcomes Reported in This Study and Selected Literature^a^

study	synthesis type	number of aa	isolation & purification	yield (purity) %
So et al. (2010)^18^	Linear	5	OSN and chromatography	92 (94)
Yoe et al. (2021)^20^	Linear	10	OSN and precipitation	75 (84)
Okada et al. (2019)^16^	Linear	10	precipitation & chromatography	65 (99)
Bisht et al. (2019)^38^	Linear	16	precipitation, extraction and chromatography	35 (crude)
				14 (HPLC)
Takahashi et al. (2017)^14^	Linear	20	solvent extraction	73 (84)
Okada et al. (2013), Bivalirudin^26^	6 fragments (LP)	20	precipitation & chromatography	85 (crude)
Okada et al. (2013), h-Ghrelin^26^	6 fragments (LP)	28	precipitation & chromatography	68 (crude)
Liu et al. (2020)^27^	6 fragments (LP)	31	precipitation & chromatography	52 (crude)
				28 (HPLC)
Schneider et al. (2005)^28^	3 fragments (SP)	39	precipitation & chromatography	30 (60)
Frederick et al. (2021)^29^	4 fragments (SP)	42	OSN & chromatography	46 (70, crude)
**this study**	**8 fragments (SP)**	**39**	**OSN only**	**88 (74)**
Jalan et al. (2026)^31^	2 Fragments (SP)	42	TFF & chromatography	45 (93%)
Han et al. (2025)^32^	2 Fragments (SP)	42	chromatography	48 (HPLC)

a OSN, organic solvent nanofiltration. (SP) indicates fragments were made via solid-phase synthesis, while (LP) indicates fragments made via liquid-phase synthesis. TFF, Tangential flow filtration.

**Figure 11:**
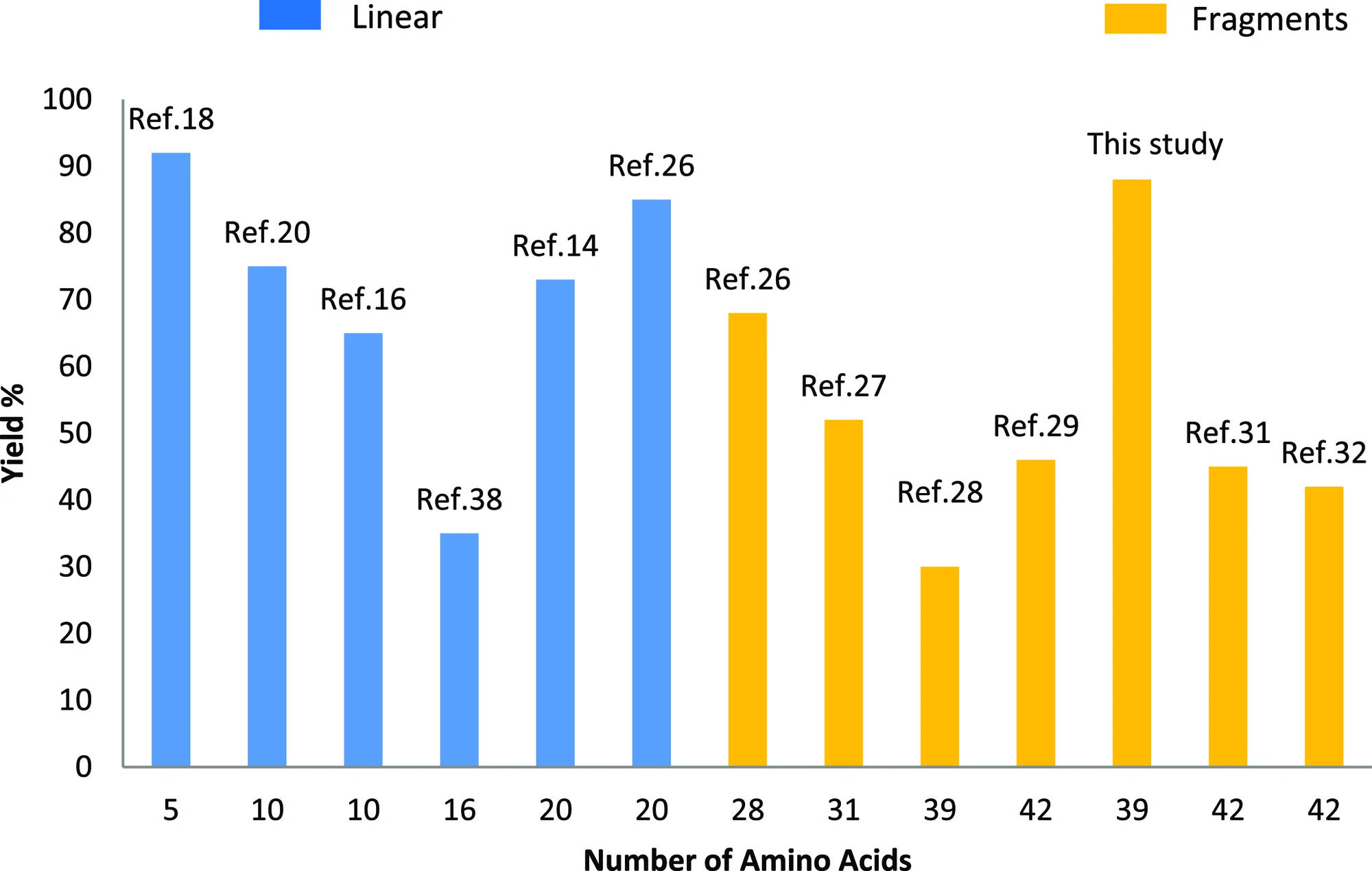
Comparison of crude yields reported in this study with other LPPS-based peptide syntheses.

A notable limitation in previous studies has been the significant loss of yield during chromatographic purification (Table 3). This issue was successfully mitigated in the current work by employing an OSN-based purification approach, which minimized product loss.

Overall, the findings highlight that the strategy described here represents a promising and efficient alternative for synthesizing longer peptides. It achieves high crude yields and maintains competitive purity levels, while reducing dependence on chromatography and the associated yield loss.

## Conclusion

This study represents a breakthrough in nanostar sieving, demonstrating the effectiveness of the PEPSTAR method for the efficient synthesis of a 39-mer peptide via fragment condensation. We established that Nanostar **3** enhances solubility and is efficiently retained by the PBI membrane, enabling the effective removal of excess peptide fragments after each coupling cycle. This significantly improves both synthesis efficiency and final product purity.

Furthermore, the PEPSTAR system proved amenable to automation, enabling unattended operation while maintaining precise process control. The integration of a photolabile linker facilitated easy cleavage of peptide intermediates, improving reaction monitoring and optimization.

We also successfully demonstrated the application of nanostar sieving with polymeric membranes for peptide isolation, eliminating the need for chromatographic purification. This streamlines the purification process, reducing both solvent consumption and processing time.

These findings highlight the potential of PEPSTAR as a scalable approach to peptide synthesis. By eliminating the need for chromatographic purification and minimizing solvent usage during convergent fragment condensation, once suitably pure fragments are available, this method enhances both efficiency and environmental sustainability. Future efforts will focus on refining membrane selectivity to enable one-step purification in organic solvents and incorporating highly pure synthetic fragments to maximize yield and prevent side reactions. These advancements will further solidify PEPSTAR as a viable alternative for large-scale pharmaceutical peptide production.

## Methods

### Materials

Starting materials were obtained from commercial suppliers and used without further purification unless stated otherwise: 4-arm PEG-NH_2_ 10KDa (≥95%), 8-arm PEG-NH_2_ 10KDa (≥95%), 8-arm PEG-NH_2_ 20KDa (≥95%) from Sinopeg. *N*,*N*′-diisopropylcarbodiimide (DIC, 99%), hydroxybenzotriazole (HOBt, ≥97%), piperidine (ReagentPlus, ≥99%), trifluoracetic acid (TFA, ReagentPlus, ≥99%) from Sigma-Aldrich. Triisopropylsilane (TIPS, ≥98%), 4-bromomethyl-3-nitro benzoic acid (97%), 1,4-sithiothreitol (97%), and *N*-(9-fluorenylmethyloxycarbonyl)­oxysuccinimide (Fmoc-OS, 97%) from Manchester organics. 4-[(2,4-dimethoxyphenyl)­(Fmoc-amino)­methyl]-phenoxyacetic acid (Fmoc-Rink linker) (≥98%) from TCI.

Solvents were obtained from commercial suppliers and used without further purification, except where specified.

Peptide fragments were used as received from *Eli Lilly and Company* without any further purification: Fragment 1 (96.57%), Fragment 2 (97.65%), Fragment 3 (98.44%), Fragment 4 (98.49%), Fragment 5 (97.33%), Fragment 6 (97.23%), Fragment 7 (99.01%), Fragment 8 (97.53%).

General information about membranes and the peptide synthesizer can be found in the Supporting Information (general information).

## Characterization Methods

### Nuclear Magnetic Resonance (NMR)

NMR spectra were obtained at 298 K, using either a Bruker AV­(III)­400 spectrometer equipped with a BBFO probe or Bruker AV­(III)­400HD spectrometer equipped with a nitrogen cryoprobe operating at frequencies of 400 MHz (1H) and 101 MHz (13C). The spectra were measured as dilute solutions in CD_3_OD. Shifts are expressed in parts per million (ppm) downfield with the solvent residual peak (CD_3_OD δH 3.31, δC 49.0) as the internal standard.

### Ultra-High Performance Liquid Chromatography with Mass Spectrometry (UPLC-MS)

Analysis was performed on an Agilent 1290 Infinity ll UPLC system equipped with a UV–visible diode array variable wavelength detector (DAD), an evaporative light scattering detector (ELSD), and coupled to Agilent 6130 single quadrupole mass spectrometer (MS). The ELSD was set to a gas flow rate of 1.6 SLM, with nebulizer temperature of 55 °C and evaporator temperature of 45 °C. MS was set to a gas flow rate of 8.0 L/min, nebulizer pressure at 35 psi, gas temperature of 350 °C and capillary voltage of 4000 V. Both positive and negative ion modes were obtained from electrospray ionization. *ChemStation* was used to process the data, and *OriginPro 2024b* to visualize the data. All samples were filtered using syringe filter, Polytetrafluoroethylene (PTFE) membrane, 0.2 μm before UPLC analysis. Different methods were used for analysis depending on sample properties (see UHPLC methods in Supporting Information).

### DrySyn Illumin8MkII 365 nm Photoreactor

Samples were taken after each coupling cycle and diluted with acetonitrile or methanol and placed in the photoreactor provided with 8 UV LEDs emits a wavelength of 356 nm for 10–15 min for photocleavage in this study. Data of photocleaved products can be found in the Supporting Information (Table S1, Figures S6 and S7).

## Purification Methods

### Flash Chromatography Using Biotage Selekt

Purification of the synthesized photocleavable linker were performed using reversed phase flush chromatography using biotage Sfär reversed phase C18 D, 100 Å, 30 μm column. The mobile phase was a binary system comprised of deionized water buffered with 0.1% formic acid (Solvent A) and acetone buffered with 0.1% formic acid (Solvent B). Following gradient of B was developed for analysis: 40–40%—3 column volume (CV), 40–95%—12CV, 95–95%, 5CV. The flow rate was set at 75 mL/min, and the eluent was monitored at 230 nm, and 260 nm.

## Membrane Purification

Membrane purification will be mentioned in the peptide synthesis part, and UHPLC data for the purified peptide-bound support can be found in the Supporting Information (Diafiltration data, Figure S3).

## Synthesis

Reactions were performed in an oven-dried apparatus under argon atmosphere using standard Schlenk-Lines. Solvents were removed using a Büchi rotary evaporator at 35 °C, or evaporation under stream of Nitrogen. Reactions were monitored by UPLC-MS.

## Experimental Procedures

### Synthesis of the Photolabile Linker, 4-(((((9*H*-Fluoren-9-yl)­methoxy)­carbonyl)­(butyl)­amino)­methyl)-3-nitrobenzoic acid, (**4**) (Scheme S1 in the Supporting Information)

4-bromomethyl-3-nitro benzoic acid (10 g, 38.6 mmol) was dissolved in acetonitrile (40 mL) and added dropwise to *n*-butylamine (14.0 g, 193 mmol, 19 mL) at 5 °C and the reaction mixture was stirred for 16 h at room temperature. The volatiles were evaporated at 30 °C and the residues were dissolved in dioxane/10% aq. Na_2_CO_3_ 1:1 (100 mL) and stirred for 30 min. The mixtures was then concentrated to half at 30 °C before it was cooled to 5 °C in ice bath and (19.6 g, 57.9 mmol) *N*-hydroxysuccinimyl (9H-fluoren-9-yl)­methoxycarbonate (Fmoc-OSu) dissolved in dioxane (125 mL) was added. The mixture was stirred for 45 min and then allowed to warm to room temperature, followed by stirring for 16 h, and the completion of the reaction was monitored by UHPLC-MS. The volatiles were removed, and the mixture was washed with diethyl ether (3 × 100 mL). The aqueous solution of the residue was acidified with concentrated HCl and extracted with ethyl acetate (3 × 100 mL). The organic layers were dried over Na_2_SO_4_, and the solvent was evaporated to obtain the crude product. Purification was performed using reversed-phase flash column chromatography on Biotage Selekt, following the previously described method. The collected fractions were analyzed by UHPLC, and the product-containing fractions were combined. After removing acetone, the product was extracted with ethyl acetate (3 × 100 mL). The organic layers were combined, dried over Na_2_SO_4_, and the solvent was evaporated to yield the pure product **4** as a pale-yellow powder (16 g, 87%), UHPLC-MS in can be found in Figure S2 in the Supporting Information.

δH (400 MHz, MeOD): 8.5 (1H, d *J* 1.5, Ar–H), 8.2 (1H, dd *J* 1.5, 8.0, Ar–H), 7.7 (2H, d *J* 7.7, Ar–H), 7.67 (2H, d *J* 7.7, Ar–H), 7.53 (1H, d *J* 8.0, Ar–H), 7.33 (2H, t *J* 7.4, Ar–H), 7.26 (2H, t *J* 7.4, Ar–H), 6.08 (2H, s, OCH_2_, Fmoc), 4.96 (5H, CH-Fmoc + H_2_O), 4.82 (2H, s, Ar–CH_2_), 3.25 (2H, t *J* 7.4, NCH_2_, butyl), 1.5 (2H, p. *J* 7.5, CH_2_), 1.28 (2H, h. *J* 7.5, CH_2_), 0.88 (3H, t *J* 7.5, CH_3_).

δC (101 MHz, MeOD): 172.23 (CO), 165 (CO), 149.5 (ArC), 144.9 (ArC), 141.36 (ArC), 139.45 (ArC), 139.26 (ArC), 139.1 (ArC), 134.6 (Ar–H), 129.8 (2 × Ar–H), 129.4 (Ar–H), 128.14 (2 × Ar–H), 126.4 (ArC), 122.0 (2 × Ar–H), 120.6 (2 × Ar–H), 108.2 (OCH_2_, Fmoc), 49.85 (ArCH_2_), 49.3 (C–H, Fmoc), 48.67 (NCH_2_), 31.98 (CH_2_), 21.26 (CH_2_), 14.36 (CH_3_).

**MS** (ESI^+^): calculated for C_27_H_26_N_2_O_6_ + H^+^, [M + H]^+^: 475.19 found *m*/*z*; 475.25.

### Synthesis of the 39-Mer Peptide (**9**), (Scheme S2 in the Supporting Information)

#### General Procedure for Coupling of Linkers and Fragments, Fmoc Deprotection, and Diafiltration

The amounts of materials specified in this procedure are used exclusively for the coupling and deprotection of the photolabile linker. For simplicity, the same mmol/equivalents were used for all other couplings. Additionally, consistent diafiltration volumes were maintained throughout the process.

(4.0 g, 0.20 mmol) of 8-arm PEG-NH_2_ 20 kDa was dissolved in 25 mL of a 60:40 DMF/MeCN (v/v) mixture and injected into the mixing flask through the flask outlet. The pump was then switched on to circulate the solution. In a separate flask, the photolabile linker (1.138 mg, 2.4 mmol, 1.5 eq/arm) was mixed with (648 mg, 4.8 mmol, 3eq/arm) of hydroxybenzotriazole (HOBt) and dissolved in 10 mL of the same solvent mixture. To this solution, (605 mg, 4.8 mmol, 3 eq/arm, 750 μL) of diisopropylcarbodiimide (DIC) was added. The resulting mixture was stirred for 30 min. The precipitated diisopropylurea (DIU) was filtered off, and the flask was rinsed with an additional 5 mL of solvent and filtered. The filtrates were combined and injected into the synthesizer, where the reaction mixture was allowed to circulate for 6 h. The pump flow rate was maintained at 40 mL/min and the temperature at 40 °C throughout the process. After completion of the reaction, piperidine (2.1 mL, 5% v/v) was added to the synthesizer flask, and the mixture was circulated for an additional 30 min. The permeate line was then opened, and pressure was applied through the pressure regulator at 7–10 bar. The mixture was concentrated to 30 mL before the solvent tank valve was opened to maintain the diafiltration at this volume. After 300 mL of solvent had passed through the membrane into the permeate flask, the autoshut system switched off the pump, stopping the diafiltration at 10 diafiltration volumes. Following the coupling of the final fragment (F8), the diafiltration solvent was switched to 50:50 MeOH/MeCN, and the diafiltration was performed directly after coupling, without adding piperidine. Once 10 diafiltration volumes had passed, the synthesizer contents were drained into a 500 mL flask. The synthesizer was then washed twice with 50:50 MeOH/MeCN, and all washes were collected in the same 500 mL flask. The volatile solvents were evaporated, yielding the nanostar-peptide as a thick, brown, gelatinous material (11.8 g).

## Global Deprotection and Isolation of the 39-Mer Peptide

1.0 g of the obtained dry nanostar-peptide from the synthesis was dissolved in 30 mL of the deprotection cocktail 93:3:3:1 TFA/H_2_O/triisopropylsilane/dithiothreitol (v/v/v/w) and stirred at room temperature for 3 h. The volatiles were removed under a stream of nitrogen gas, and the concentrated remaining residue was dissolved in acetonitrile (1 mL), then added dropwise to cold methyl *tert*-butyl ether (MTBE, 40 mL) in a 50 mL centrifuge tube. The precipitate was collected by centrifugation, the supernatant was discarded, and the precipitate was washed twice with MTBE before being collected again. The collected precipitate was dissolved in 59:40:1 H_2_O/IPA/TFA (v/v/v) and the resultant solution was transferred to the synthesizer and allowed to circulate. The temperature was maintained at 20 °C using a heat exchanger supplied with tap water. The cell was equipped with a 30 kDa cutoff PES membrane. The permeate line was opened, and pressure was applied through the regulator to push the solution through the membrane. The solution level was maintained at 30 mL by compensating for solvent loss from the solvent tank. After five diafiltration volumes (150 mL), all the peptides had permeated into the permeate flask. The permeate was neutralized with 2v/v % triethylamine (3 mL) and stored in the freezer at −20 °C until needed. The PES membrane was removed, and the synthesizer was washed with methanol. A 1 kDa cutoff PPA membrane was installed and washed with methanol from the solvent tank, allowing 200 mL of methanol to pass through membrane before starting the process. The peptide solution was warmed to 20 °C and then transferred to the reaction loop vessel. The temperature was maintained at 20 °C, the permeate line was opened, and the solution was pushed through the membrane using a pressure regulator set to 7 bar. After ten diafiltration volumes (300 mL) had passed through the membrane, most of the solvents were replaced with methanol from the solvent tank. The contents of the synthesizer were then transferred into a 250 mL flask, and the methanol was evaporated to yield the 39-mer peptide as a white powder (455 mg, 88%) yield calculation details can be found in the Supporting Information (Yield calculations).

## Supplementary Material



## Notes

Additional experimental procedures, characterization data, mass spectra, NMR spectra and chromatograms are provided to support the findings of this study (PDF) (PDF)
