# Supplementary material for: Toward Chromatography-Free Convergent Peptide Synthesis via Nanostar Sieving
Source: Org Process Res Dev. 2026 Aug 17;30(9):2454–68. doi: 10.1021/acs.oprd.6c00123 (PMC13595908; doi:10.1021/acs.oprd.6c00123)

Towards Chromatography-Free Convergent Peptide Synthesis via Nanostar Sieving

Mustafa Al-Kadhimi[a], James Botwright[a], Adam Oxley[b], Seok Ju Han[a], Nil Tandogan Gray[c], Michael O. Frederick[c], Ludmila Peeva*[a] and Andrew G. Livingston*[a]

[a] Dr M. Al-Kadhimi, J. Botwright, Seok Ju Han, Dr L. Peeva, Prof A. G. Livingston
School of Engineering and Materials Science
Queen Mary University of London
Joseph Priestley Building, Mile End Road, London, E1 4NS, UK
E-mail: a.livingston@qmul.ac.uk

[b] Dr Adam Oxley
Exactmer Ltd
Uk Business and Technical Park, London, Dagenham RM10 7FN

[c] Dr N. Tandogan Gray, Dr M. O. Frederick
Synthetic Molecule Design and Development
Eli Lilly and Company
Lilly Corporate Centre, Indianapolis, IN 46285, USA

*Corresponding author

Supporting information

Contents

1. General information 0
2. Synthesis 2
Synthesis of the photolabile linker 4 2
Synthesis of the 39-mer Peptide 3
3. Data and analysis 4
The photolabile linker 4
Diafiltration data 4
Photocleavage data 6
Isolated peptide data 10
4. Yield calculations 14
5. NMR spectra 15
Appendix 20

1. General information

17% polybenzimidazole (PBI) membranes and 12% polyetheretherketone (PEEK) membranes were made in our laboratory, 10 kDa, and 30 kDa cutoff polyethersulfone (PES) membranes were obtained from Biomax, and 1kDa cutoff polypiperizine amide (PPA) membranes were obtained from STERLITECH.

A dual-piston AZURA® pump (Knauer), equipped with a pressure sensor and a stainless-steel pump head, was used. The pressure sensor was preset to a maximum of 20 bar to automatically shut down the pump in case of pressure buildup due to blockage. The pump supports flow rates of up to 50 mL/min. A MiniMem membrane cell with a built-in stirrer, heat exchanger, and speed sensor was purchased from PS-Prozesstechnik GmbH. A homemade auto-shut system equipped with two solvent detectors was developed at Queen Mary University of London. A pressure regulator (R3A series) was sourced from Swagelok®.

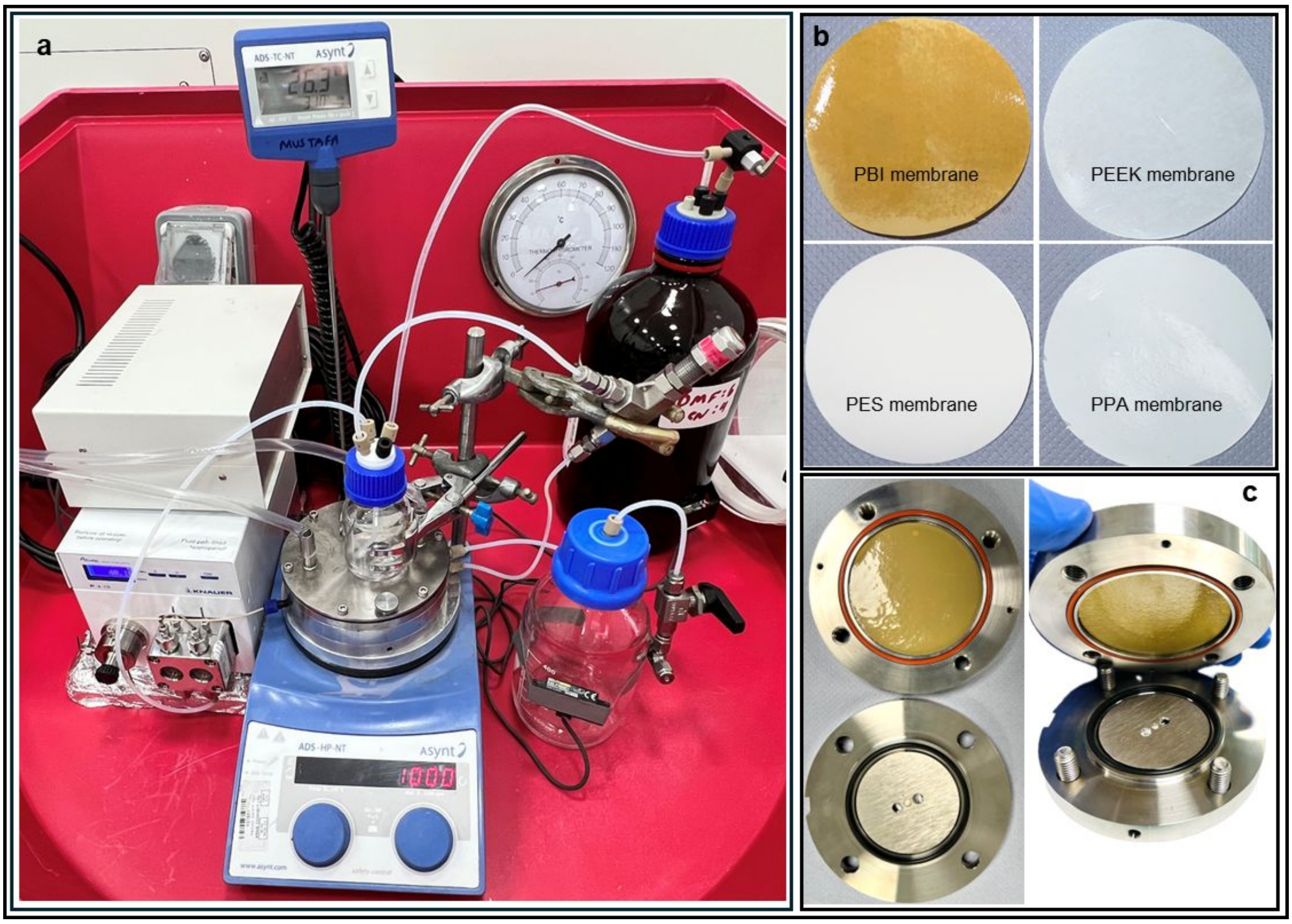

Figure S1. (a) The peptide synthesizer. (b) Various membrane types used for reactions and peptide isolation. (c) An inside view of the membrane cell, showing the PBI membrane and the cell stirrer.

Centrifuge 5702 R purchased from Eppendorf. For this study the max speed was set to 4400 round per minute (RPM), the temperature at 20 °C and the time at 30 min.

UHPLC methods

Method 1 was used for the monitoring the synthesis and purification of the photolabile linker. **Method 2** was used for monitoring couplings, deprotection, and diafiltrations during the peptide synthesis. **Method 3** For analysis during photocleavage of the peptide species. **Method 4** for the analysis of the deprotected and isolated 39-mer peptide.

Method 1: A reversed-phase C18 Column, 1.7 µm, 2.1 mm X 50 mm, for separation. The mobile phase for UPLC was a binary system comprised of deionized water buffered with 0.1% formic acid (Solvent A) and 50:50 MeCN/MeOH (Solvent B). Following gradient of B was developed for analysis: 0 min – 20%, 5 min – 95%, 7 min – 95%, 8 min-20%. The UPLC pump flow rate was set at 0.3 mL/min, and the column temperature was kept at 50°C. The eluent was monitored at 230 nm, and 260 nm.

Method 2: A reversed-phase ACQUITY UPLC BEH C18 Column, 130Å, 1.7 µm, 2.1 mm X 100 mm, for separation. The mobile phase for UPLC was a binary system comprised of deionized water buffered with 0.1% formic acid (Solvent A) and 50:50 MeCN/MeOH (Solvent B). Following gradient of B was developed for analysis: 0 min – 20%, 4 min – 95%,

8.5 min – 95%, 9.5 min – 20%, 10 min-20%. The UPLC pump flow rate was set at 0.3 mL/min, and the column temperature was kept at 60°C. The eluent was monitored at 230 nm, and 260 nm.

Method 3: A reversed-phase ACQUITY UPLC BEH C18 Column, 130Å, 1.7 μm, 2.1 mm X 100 mm, for separation. The mobile phase for UPLC was a binary system comprised of deionized water buffered with 0.1% formic acid (Solvent A) and 50:50 MeCN/MeOH (Solvent B). Following gradient of B was developed for analysis: 0 min – 20%, 6 min – 95%, 9 min – 95%, 9.5 min – 20%, 10 min-20%. The UPLC pump flow rate was set at 0.3 mL/min, and the column temperature was kept at 60°C. The eluent was monitored at 230 nm, and 260 nm.

Method 4: Poroshell "Chiral-T" column, 2.7 μm, 2.1 mm X 100 mm, for separation. The mobile phase for UPLC was a binary system comprised of 30:70 MeOH/20 mM ammonium formate (pH 4) (Solvent A) and 50:50 MeCN/MeOH (Solvent B). Following gradient of B was developed for analysis: 0 min – 5%, 10 min – 5%, 50 min – 95%, 55 min – 95%, 55.1 min – 5%, 60min – 5%. The UPLC pump flow rate was set at 0.3 mL/min, and the column temperature was kept at 45°C. The eluent was monitored at 230 nm, and ELSD.

2. Synthesis

Synthesis of the photolabile linker 4

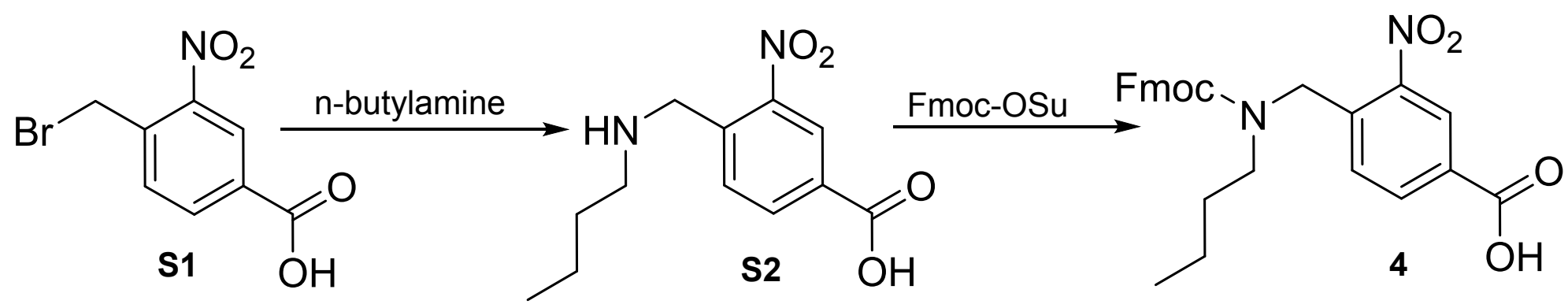

Scheme S1. The synthesis route for the photolabile linker **4**.

Synthesis of the 39-mer Peptide

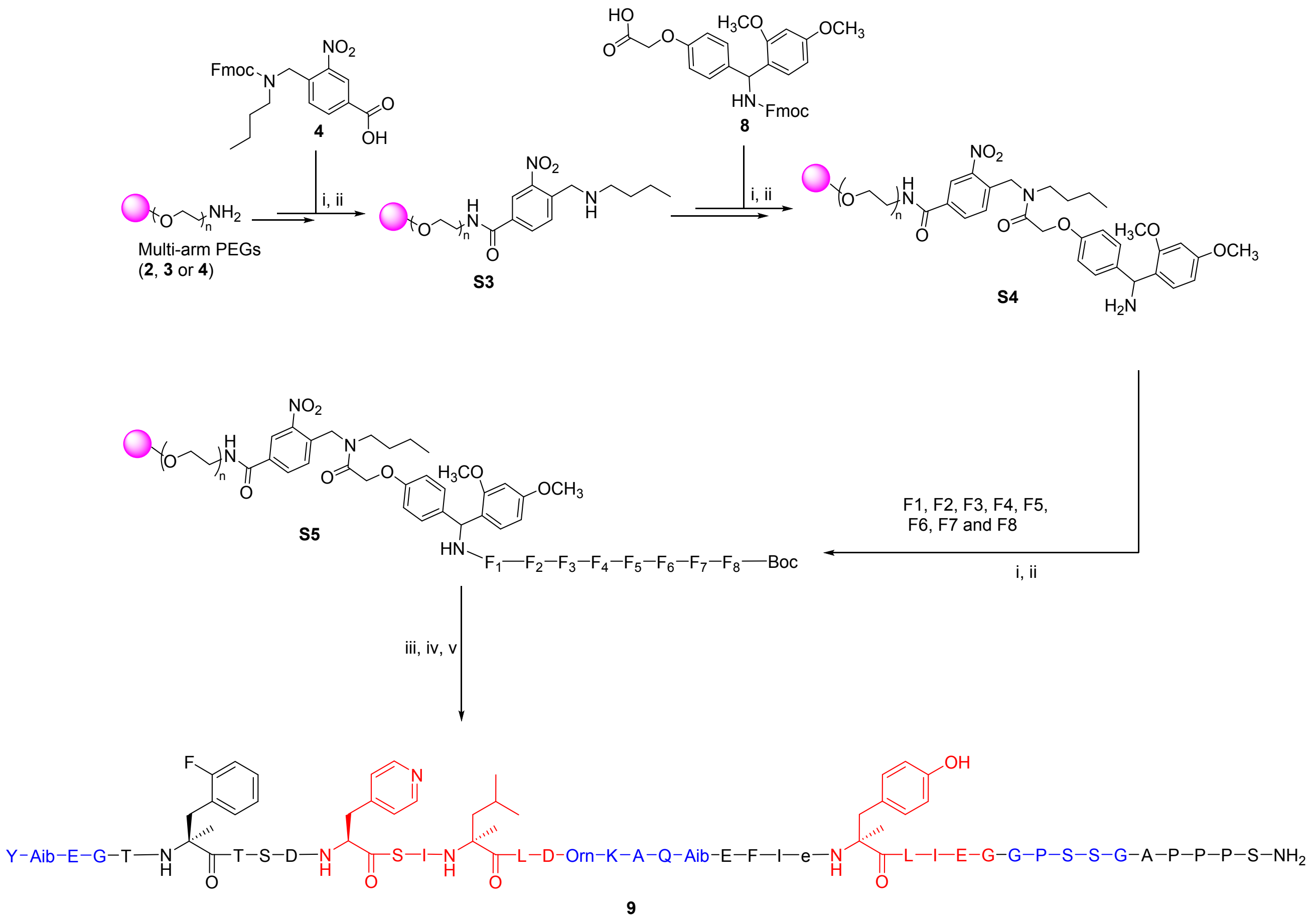

Scheme S2. The synthesis route for the 39-mer Peptide, conditions: i) 1.5 eq. (**4**, **8** or fragments)/arm, 3 eq. DIC/arm, 3eq. HOBt /arm, 60:40 DMF/MeCN, 40 °C, 6 hrs; ii) piperidine 5 v/v %, 40 °C, 30 min; iii): 93:3:3:1 trifluoroacetic acid/water/triisopropylsilane/dithiothreitol, rt, 3 hours; iv) evaporation under N_2 Stream, precipitation with cold methyl tert-butyl ether; v) isolating the 39-mer peptide using organic solvent nanofiltration.

3. Data and analysis

The photolabile linker

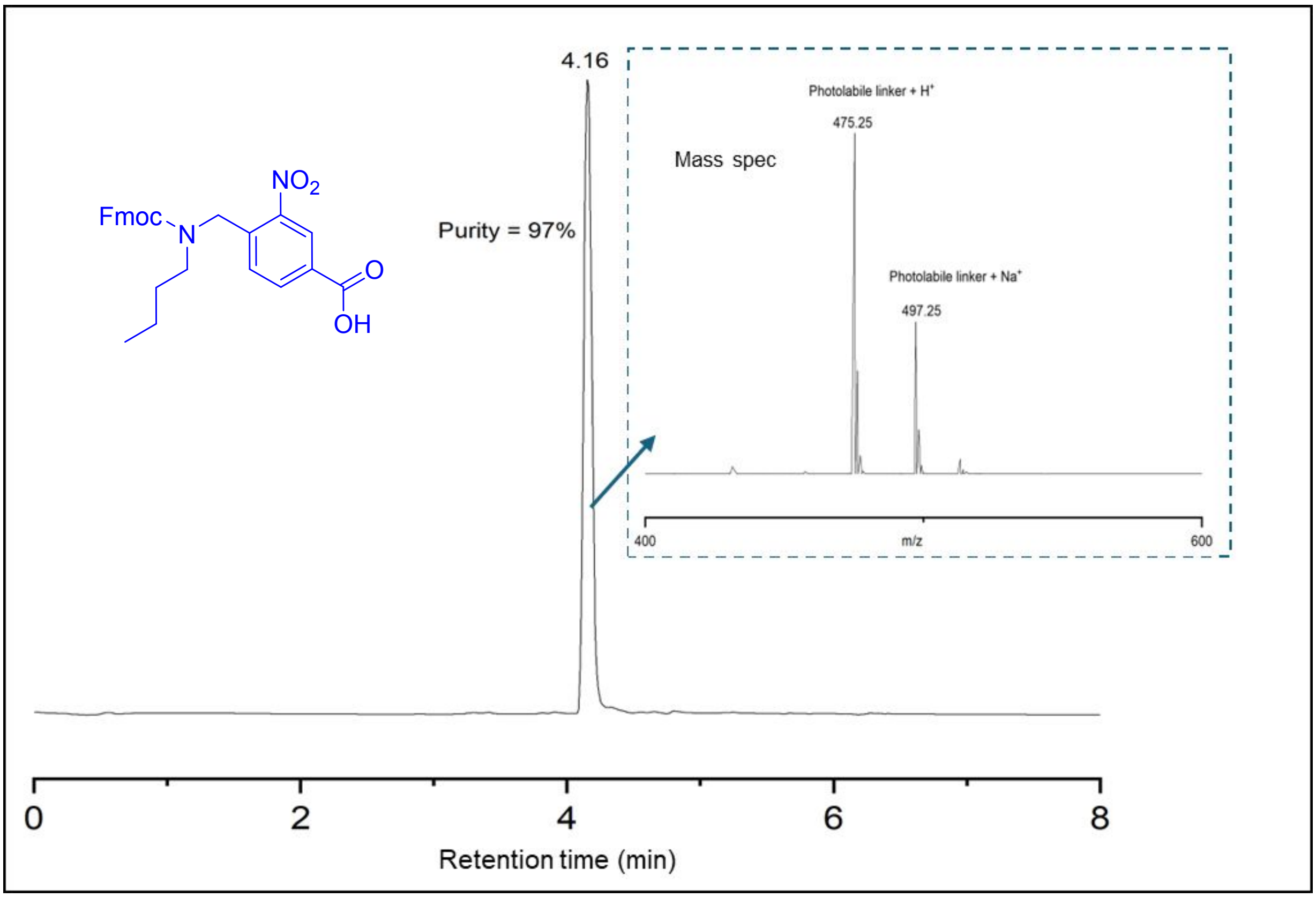

Figure S2. UHPLC-MS chromatograms (UV absorbance and the corresponding mass spec) of the photolabile linker **4**.

Diafiltration data

After 10 diafiltration volumes, UHPLC analysis confirmed the complete removal of excess fragments and reaction debris. Notably, the retention time of the purified product gradually increased with the sequential addition of peptide fragments, consistent with the expected elongation of the peptide chain (Figure S3). However, after the addition of fragment 6, the peak corresponding to the peptide-bound support became weaker and broader. This effect is likely due to the low solubility of the product in the UHPLC mobile phase and to the increased molecular weight.

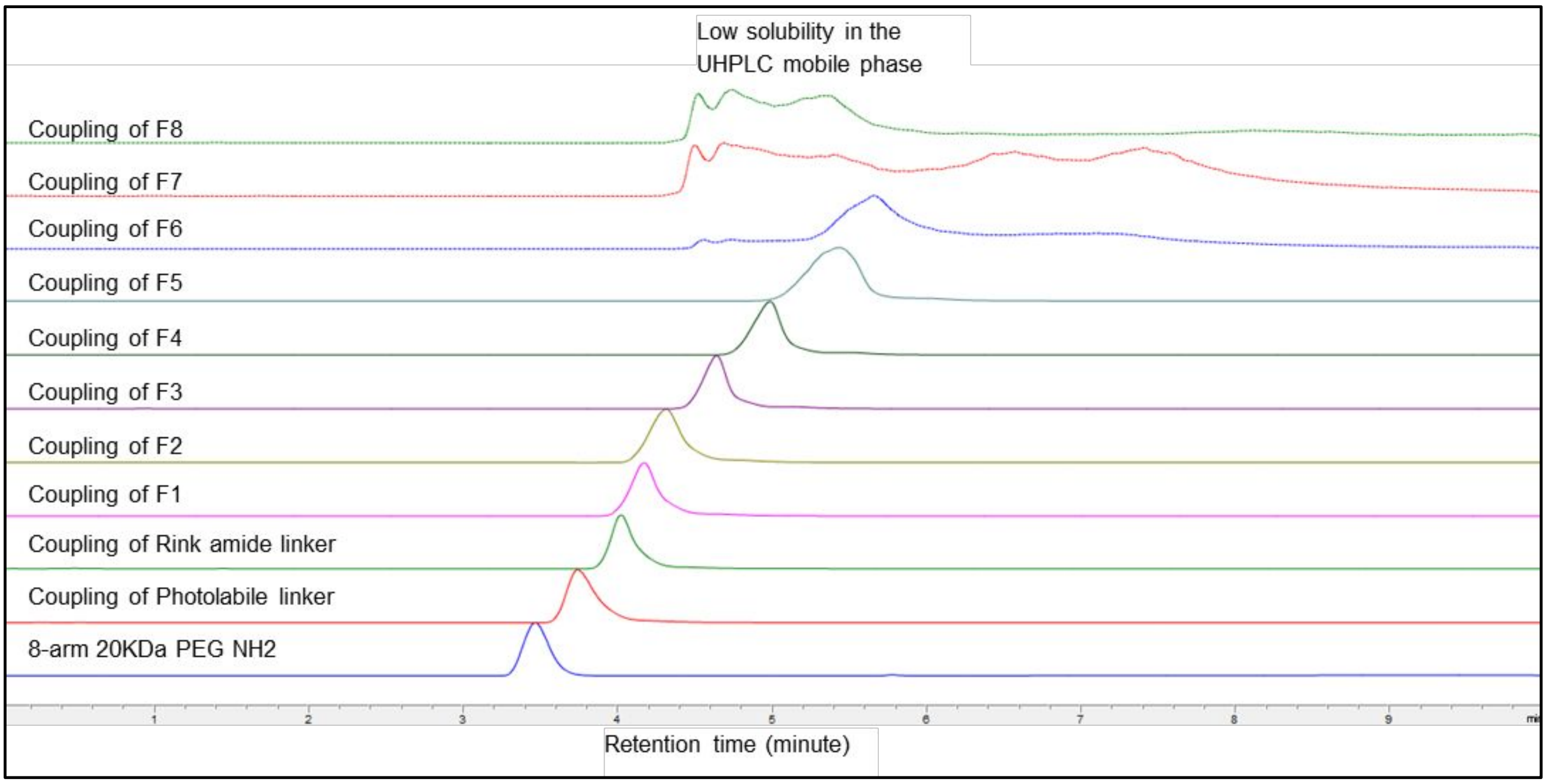

Figure S3. UHPLC chromatograms show the peptide-bound support at different cycles of the 39-mer peptide synthesis.

Gas chromatography (GC) analysis further indicated that less than 0.01% of piperidine remained after ten diafiltration volumes, ensuring minimal quenching (≤4%) of the fragment in the subsequent coupling cycle. Figure S4 demonstrates the effect of piperidine concentration on the coupling reaction. Fragment 1 was used to investigate the percentage of quenching at various piperidine concentrations: 0.01%, 0.05%, 0.1%, 0.5%, and 1% (v/v). The reactions were performed using the same conditions used in run 5, but without using nanostar and the concentration of the Fragment used is 40 mM. The results showed that piperidine concentrations of 0.5% and 1% (v/v) led to complete quenching of the activated fragment, while 0.05% resulted in 25% quenching, and 0.1% led to 68% quenching. The lowest concentration, 0.01%, resulted in only 4% quenching, which is negligible given that 1.5 equivalents of the fragment are added per arm of the nanostar.

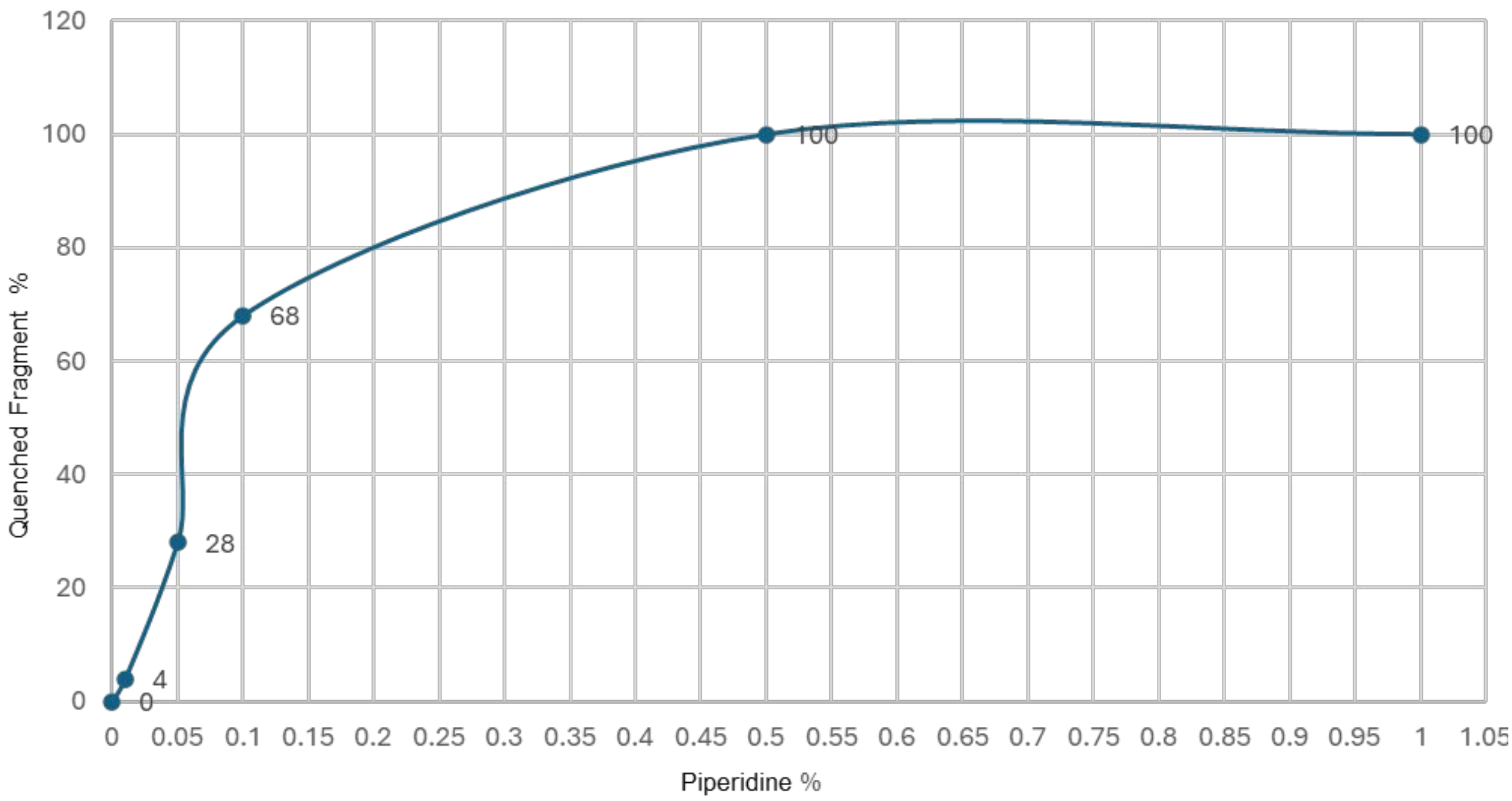

Figure S4. The effect of piperidine concentration on the peptide coupling reaction.

Photocleavage data

After each coupling and diafiltration cycle, a 10 µL sample was withdrawn from the reaction loop vessel and diluted with MeCN to a final volume of 200 µL in a UHPLC-MS vial. The sample was first analysed before photocleavage (Figure S3) and then placed in the photoreactor for 10–15 minutes before being reanalysed.

The first photocleavage was performed after the attachment of the Rink amide linker, and the expected cleaved product was compound **S6**, as shown in Scheme S3.

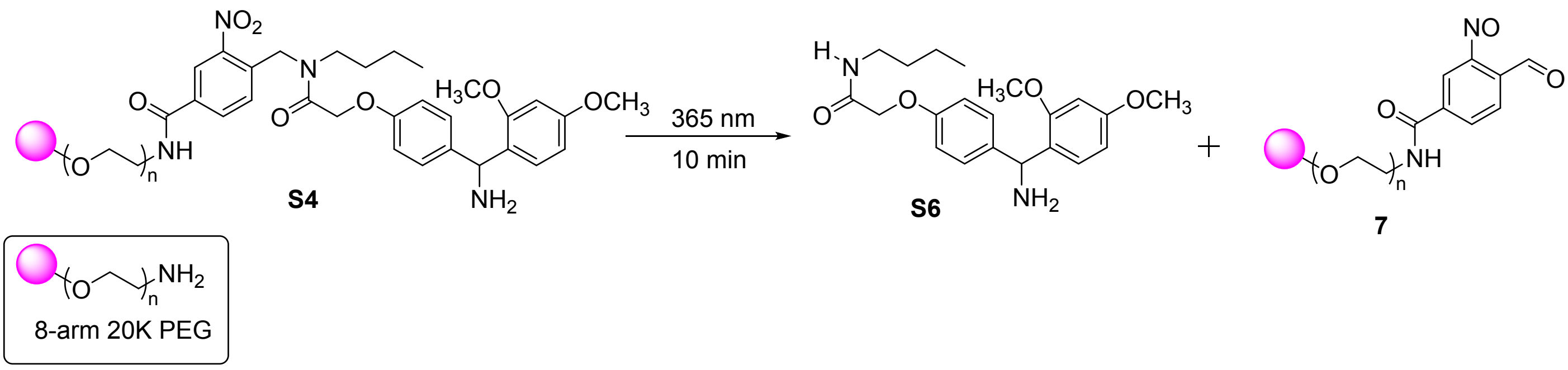

Scheme S3. The expected photocleavage product of compound S4.

However, instead of compound **S6** compound **S7** was observed, which lacked an amino group. This discrepancy is attributed to Rink amide linker fragmentation in mass spectrometry, as detailed in Scheme S4.

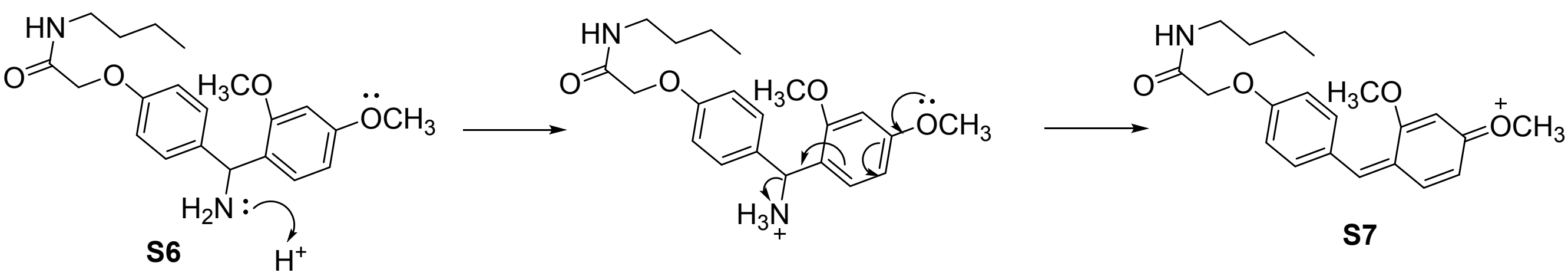

Scheme S4. Rink amide linker fragmentation in mass spectrometry.

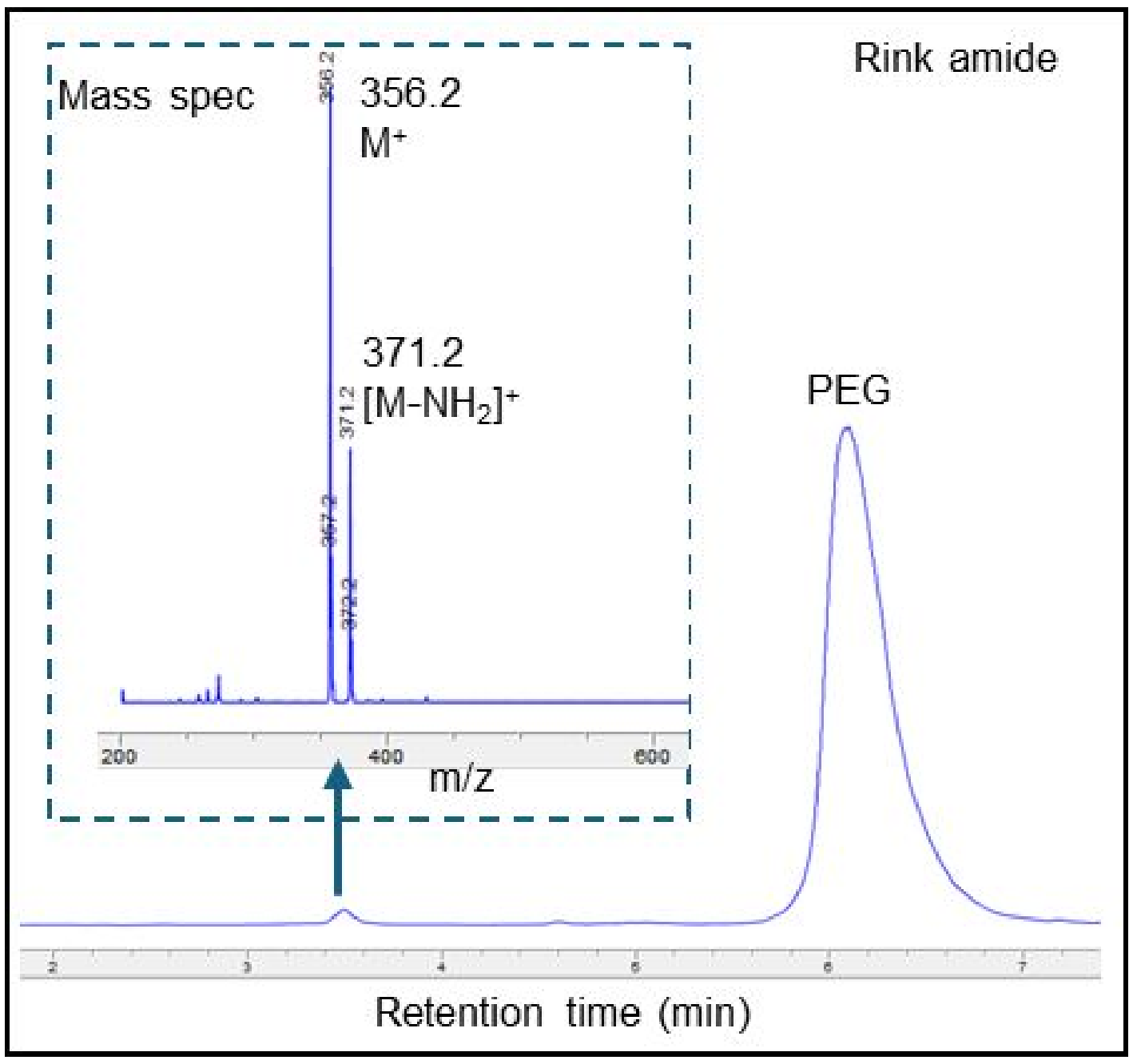

Figure S5. UHPC-MS chromatogram (ELSD and the corresponding mass spec) of the photocleaved product **S6**.

The UHPLC-MS chromatograms for each peptide coupling cycle are presented in Figure S6 and Figure S7 and the corresponding mass spectrometry data for the expected cleaved products are summarized in Table S1.

Table S1. Mass spectrometry data of photocleaved products at different stages of peptide synthesis

Photocleaved products (Appendix)	Chemical formula of cleaved product	Theoretical mass of the photocleaved product (neutral) g/mol	Observed ion (m/z) g/mol	Charge status	Calculated mass(neutral) g/mol
S6 (Rink amide)	$C_{21}H_{27}N_2O_4$+	371.2	371.2	$[M]^+$	371.2
	$C_{21}H_{26}NO_4$+	356.19	356.2	$[M-NH_2]^+$	356.2
*F1= (S8)	$C_{46}H_{67}N_7O_{10}$	877.5	878.6	$[M+H]^+$	877.6
*F1-F2 (S9)	$C_{69}H_{106}N_{12}O_{17}$	1374.8	688.6	$[M+2H]^{2+}$	1375.2
		1374.8	1375.9	$[M+1H]^{+1}$	1374.8
*F1-F3 (S10)	$C_{106}H_{165}N_{17}O_{25}$	2076.2	1039.7	$[M+2H]^{2+}$	2077.4
*F1-F4 (S11)	$C_{139}H_{215}N_{21}O_{33}$	2706.6	1355.0	$[M+2H]^{2+}$	2708.0
*F1-F5 (S12)	$C_{191}H_{287}N_{29}O_{43}$	3675.1	1226.8	$[M+3H]^{3+}$	3675.6
*F1-F6 (S13)	$C_{233}H_{356}N_{36}O_{52}$	4490.6	1124.3	$[M+4H]^{4+}$	4493.2
*F1-F7 (S14)	$C_{274}H_{422}N_{41}O_{62}$	5298.1	1324.0	$[M+4H]^{4+}$	5292.0
*F1-F8 (S15)	$C_{307}H_{472}N_{45}O_{71}$	5944.5	1488.2	$[M+4H]^{4+}$	5948.7

The photocleaved products were monitored using UHPLC-MS after coupling F1, F2, F3, and F4. The data shown in Figure S6 indicate that the expected products appeared at retention times different from PEG. Additionally, a variant of the expected product lacking a butyl group was observed at a distinct retention time. We believe this butyl group loss occurred during photocleavage, or it may be attributed to the acidic environment of the mobile phase used in UHPLC-MS.

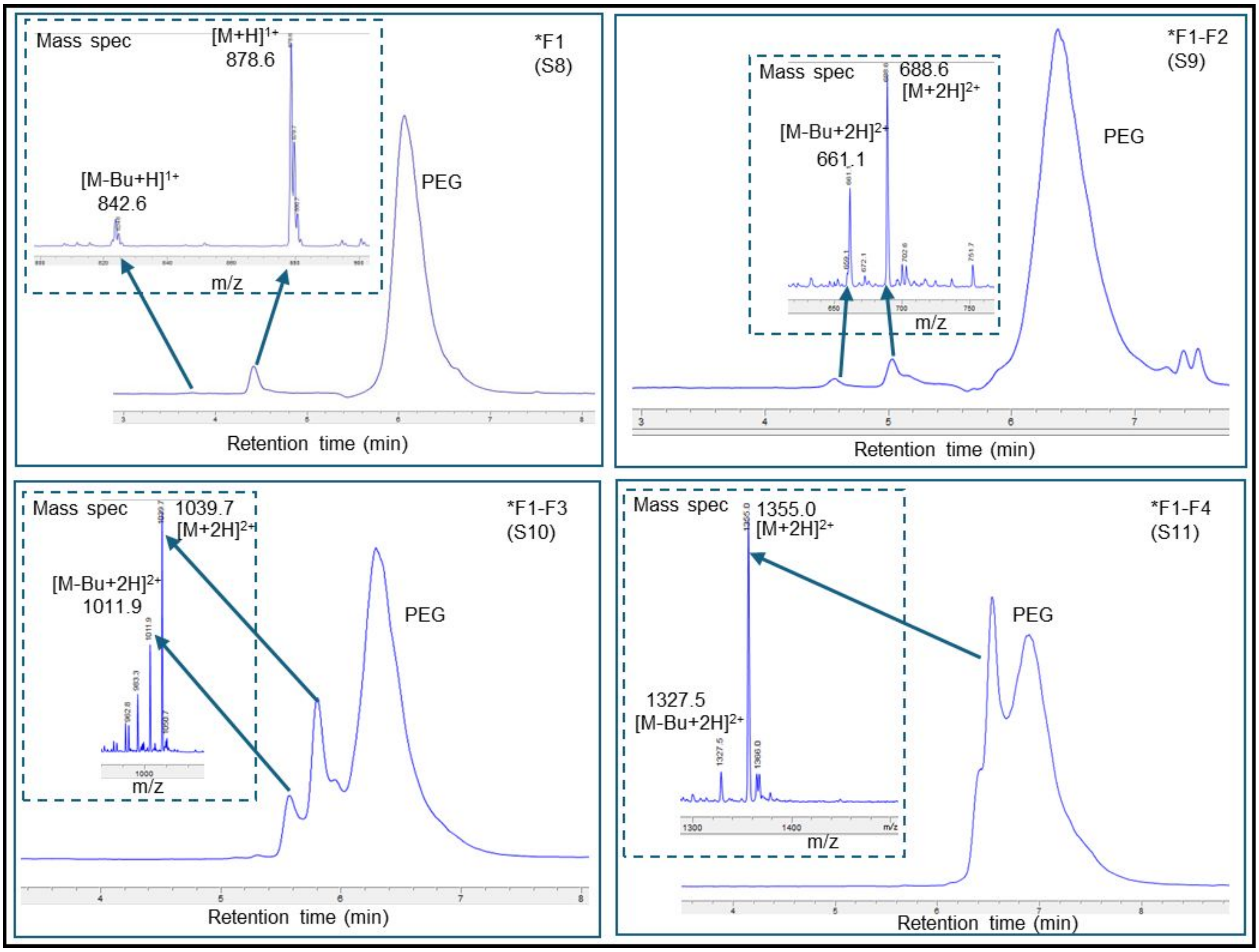

Figure S6. UHPLC-MS chromatograms (ELSD and the corresponding mass specs) of the photocleaved products; S8, S9, S10 and S11.

After the coupling of fragment 5, the photocleaved product began to co-elute with the nanostar in the UHPLC chromatogram (Figure S7), resulting in peak overlap.

In the ELSD chromatogram of the photocleaved products S14 and S15, the PEG peak appeared as a weak, broad signal due to the low solubility of the mixture in the UHPLC mobile phase. The mass peak of the photocleaved product S15 overlapped with the PEG mass peak in the mass spectrum; however, the photocleaved product mass was still detectable, confirming that the coupling of F8 had occurred.

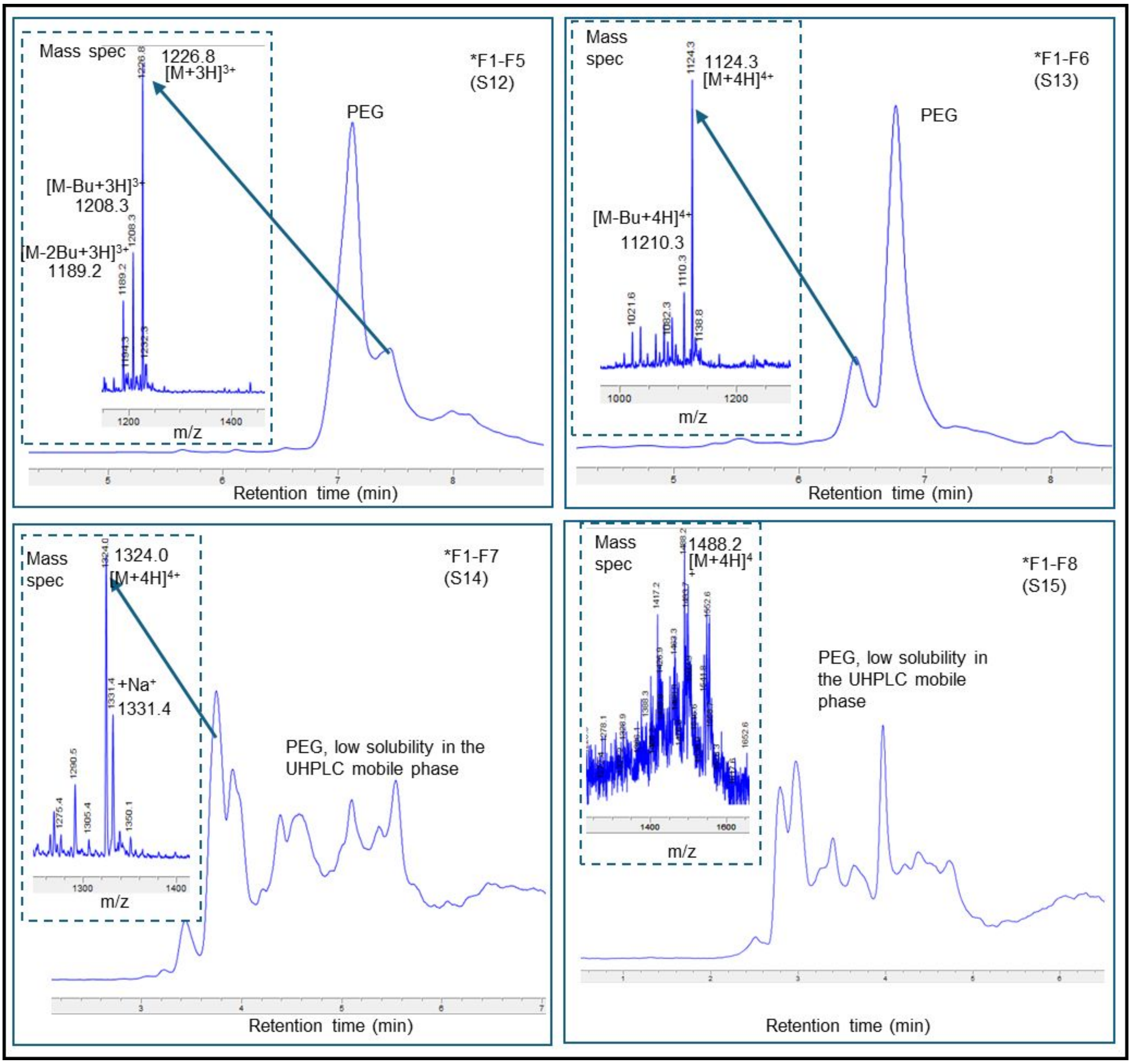

Figure S7. UHPLC-MS chromatograms (ELSD and the corresponding mass specs) of the photocleaved products S12, S13, S14 and S15.

Isolated peptide data

UHPLC-MS data from Run 5.

The peptide eluted at a retention time of 30.58 minutes with a purity of 74%. The remaining 26% consists of impurities related to fragment purity, byproducts generated during synthesis, or impurities introduced during global deprotection.

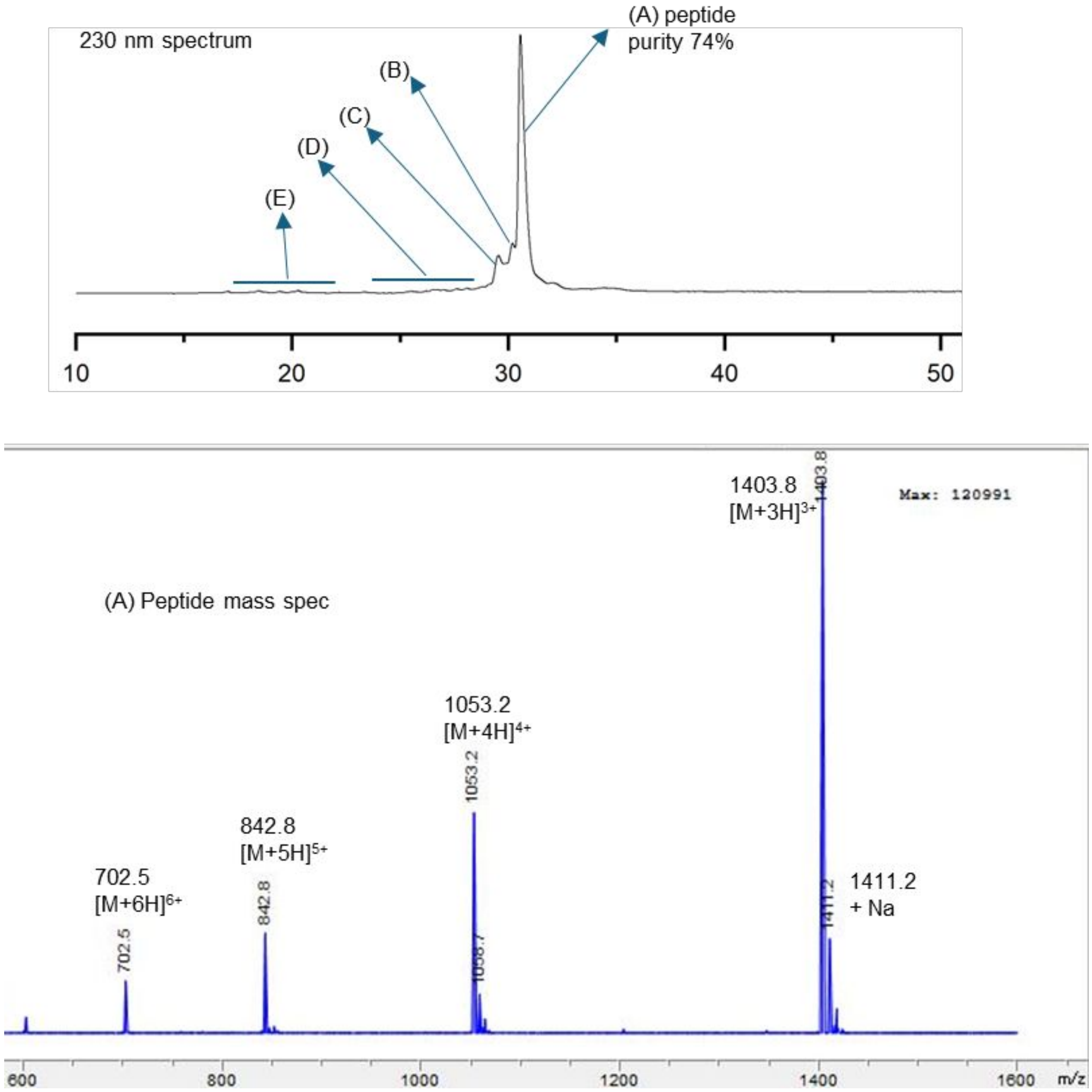

Figure S8. UHPLC-MS chromatograms (UV absorbance and the corresponding mass spec) for the isolated peptide.

In the UV chromatogram, **(B)** represents an 11% impurity, which appears to be a mixture of an epimerized peptide and a peptide missing the last N-terminal amino acid, tyrosine.

(C) accounts for 9% of the impurities, including a methylated full-length peptide, a methylated peptide missing tyrosine, and some unidentified impurities. The methylation likely occurred during global deprotection due to trace methanol used to wash the peptide-bound nanostar from the synthesizer.

Additionally, **6%** of unknown impurities were detected at various retention times (**D** and **E**). These contain ≤1% peptide missing F7 and ≤1% peptide missing F8. Such impurities may arise from the loss of protecting groups during synthesis, leading to branched peptide formation that complicates analysis, or from incomplete side-chain protection during solid-phase synthesis (Figure S9).

The actual percentage of impurities resulting from peptide synthesis is estimated to be 9.5%, while the remaining 16.5% is attributed to fragment purity, as the maximum achievable yield is estimated to be 83.5%.

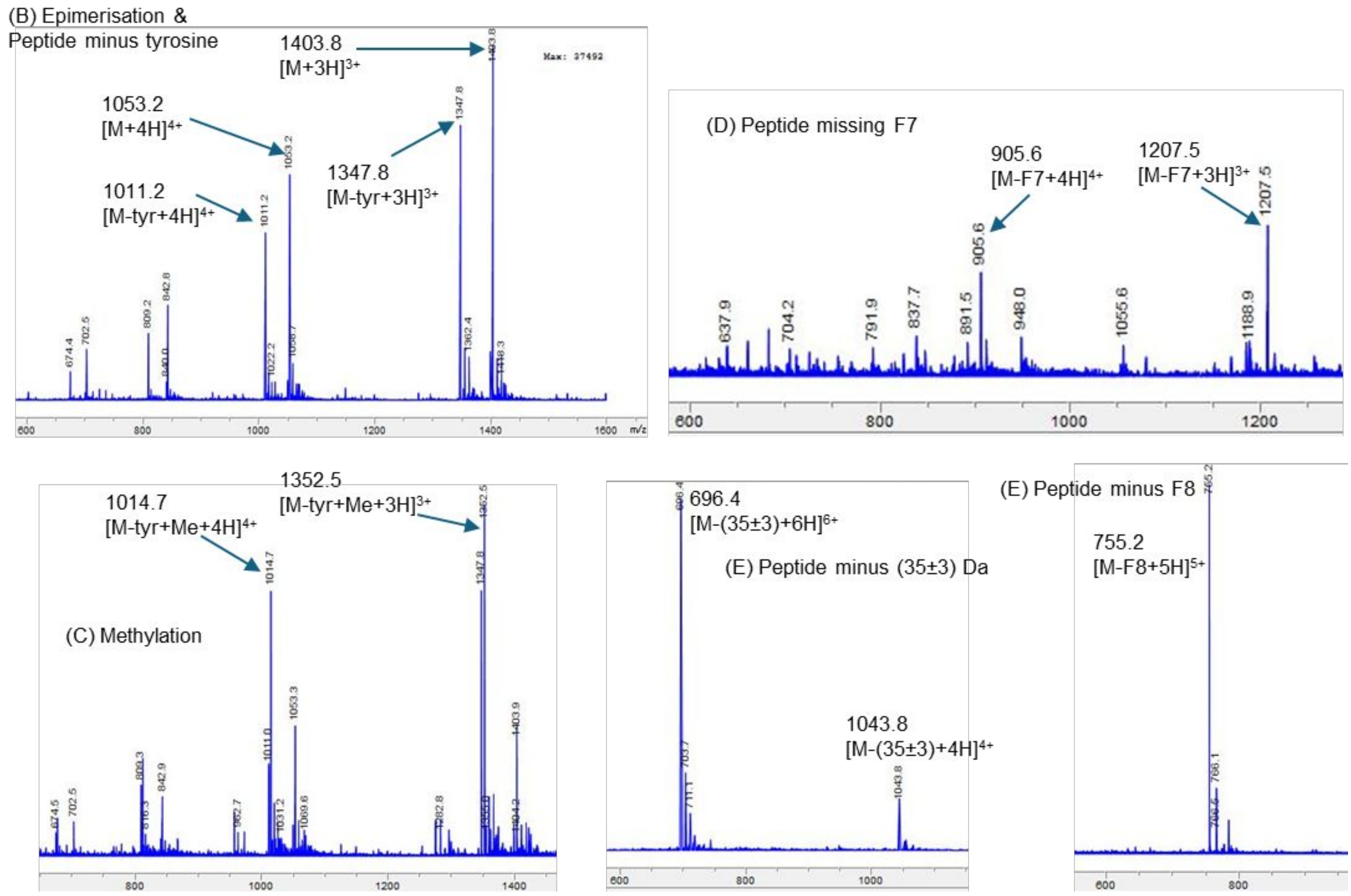

Figure S9. The mass specs of the peptide impurities.

UHPLC-MS data of Run 4.

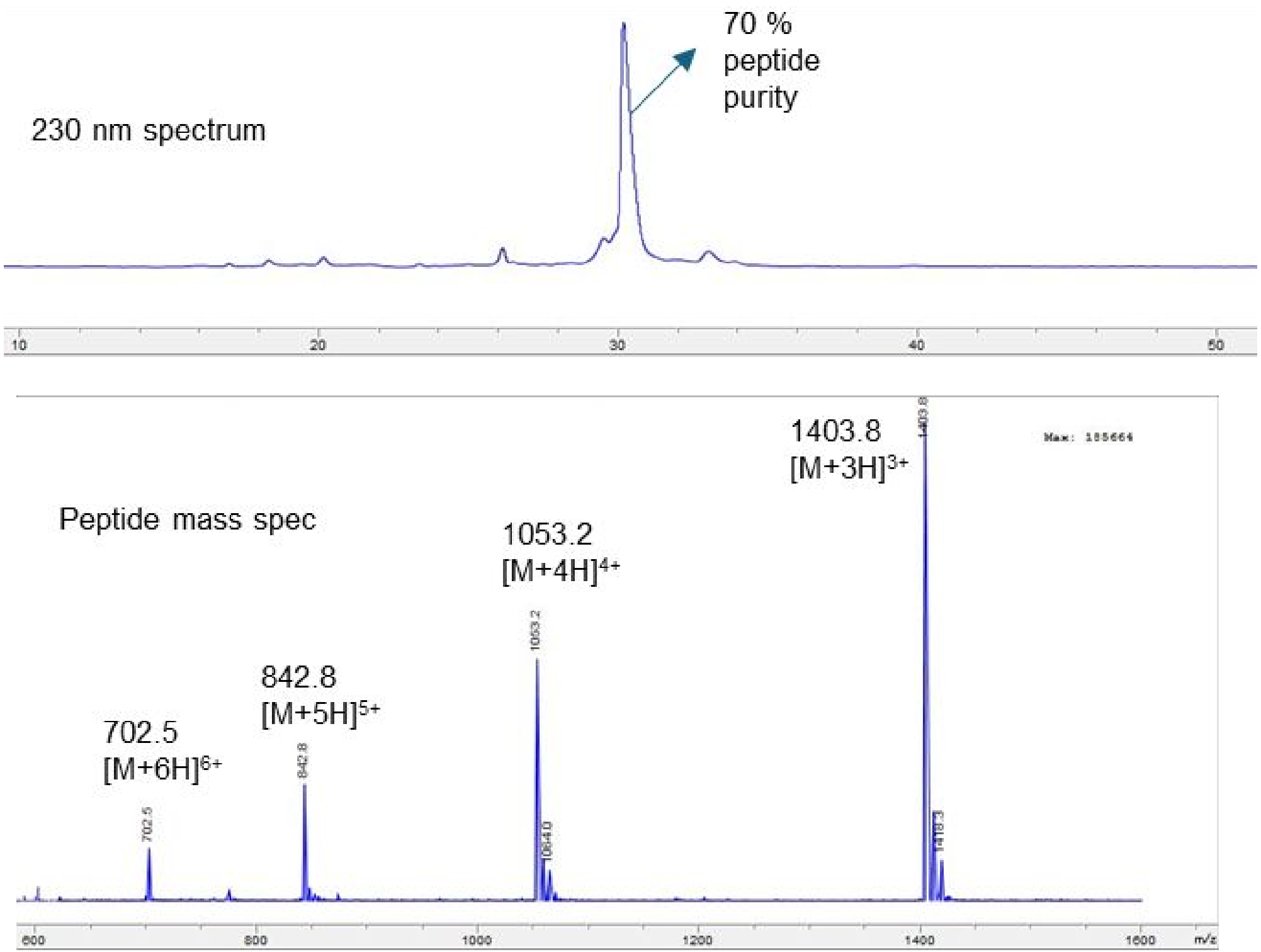

Figure S10. UHPLC-MS chromatograms (UV absorbance and the corresponding mass spec) for the isolated peptide.

UHPLC-MS data of Run 3.

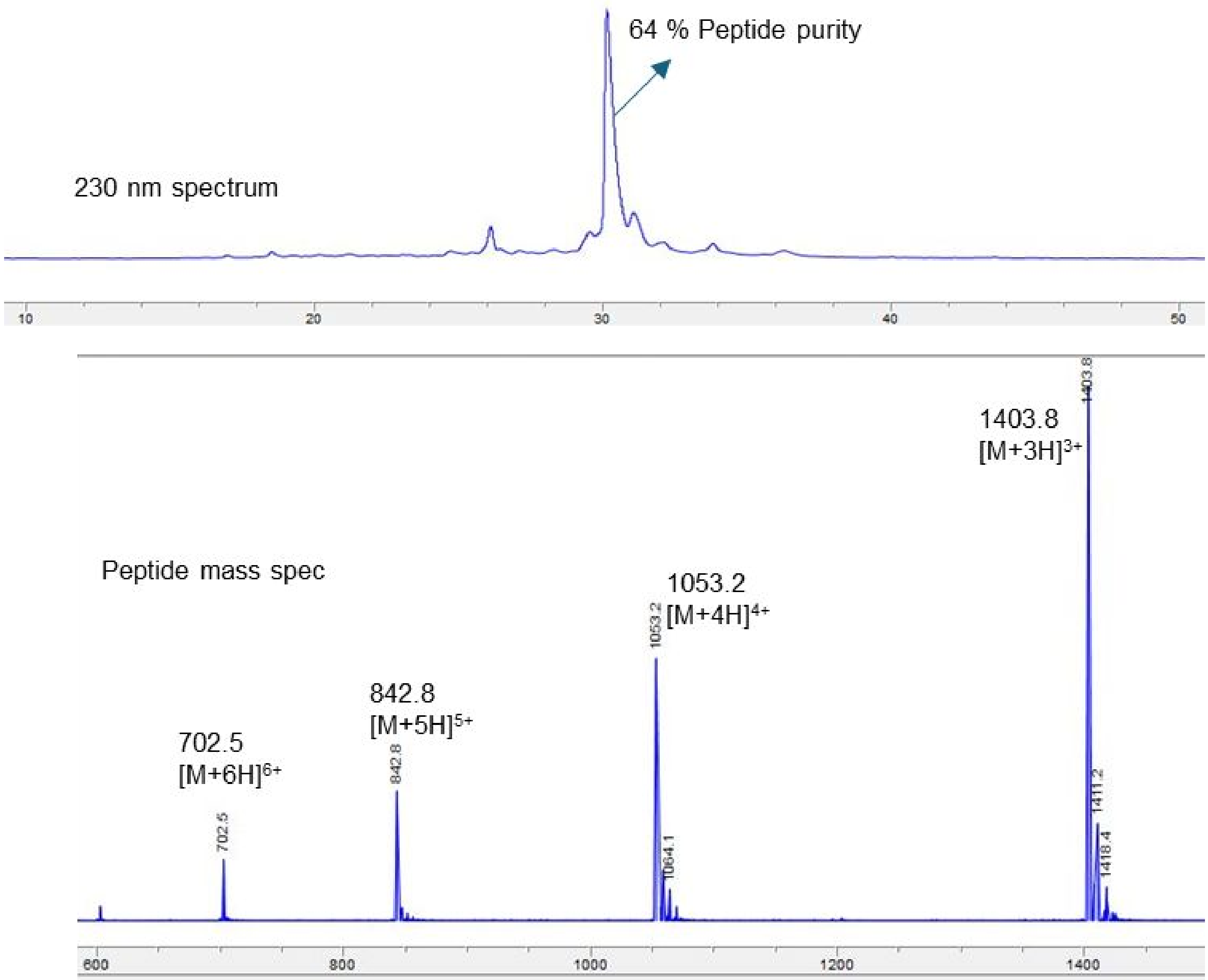

Figure S11. UHPLC-MS chromatograms (UV absorbance and the corresponding mass spec) for the isolated peptide.

UHPLC-MS data of Run 1 and 2.

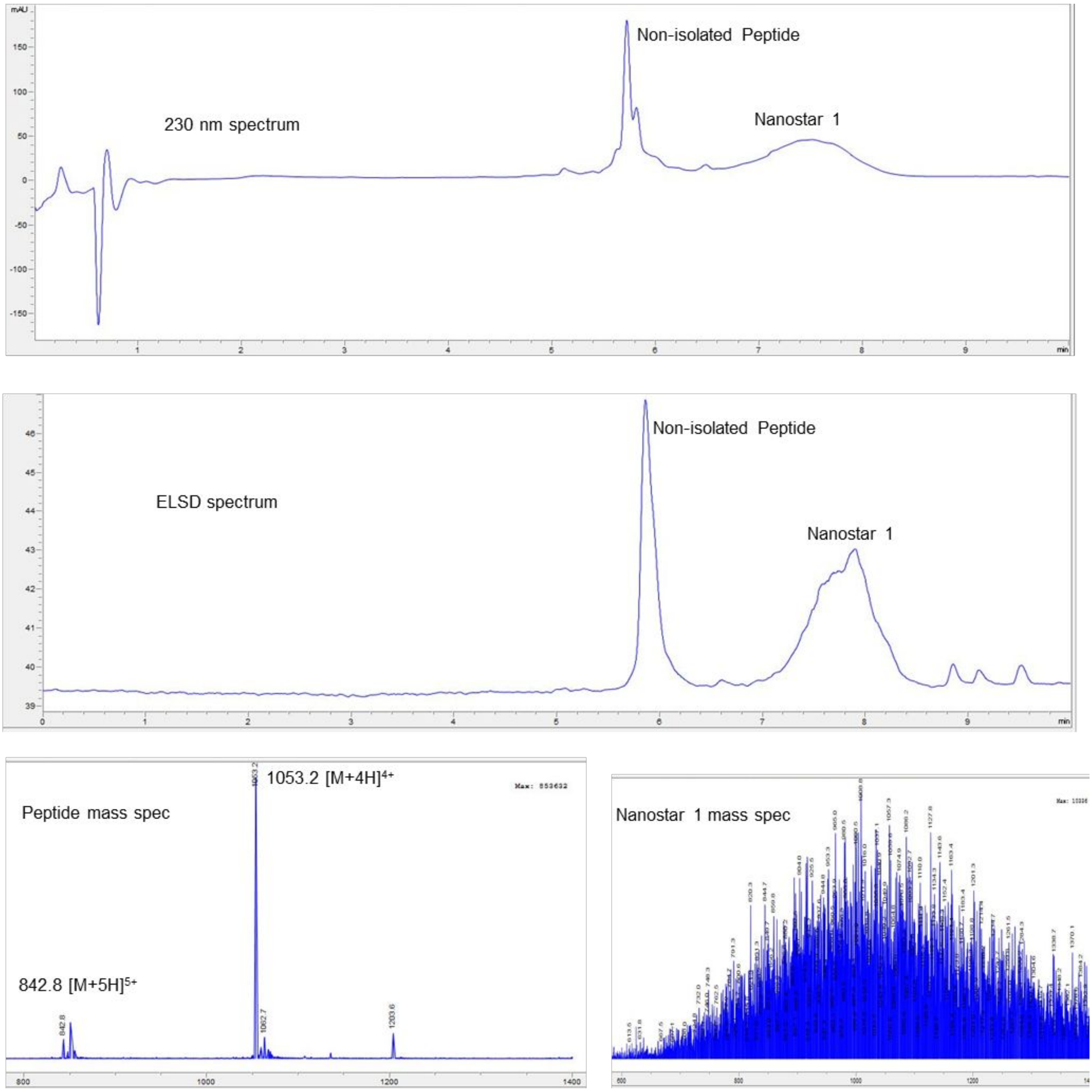

Figure S12. UHPLC-MS chromatograms (UV absorbances and the corresponding mass specs) for the peptide and nanostar mixture.

4. Yield calculations

The theoretical weight of the peptide was determined after accounting for losses due to sampling and diafiltration. The number of millimoles (mmol) of the final 39-mer peptide product was calculated based on the initial number of mmol of the nanostar, multiplied by the number of arms. For instance, when using an 8-arm 20 kDa PEG-NH_2 nanostar, the expected number of mmol of peptide is obtained by multiplying the mmol of PEG by eight. For clarity, we describe the calculation for Run 5, which used a 40 mM peptide concentration with 8-arm 20 kDa PEG-NH_2.

The molecular weight (MW) of the nanostar = 20,000 g/mol.

The number of mmol of the nanostar was calculated as follows:

mmol of nanostar = (Weight (g) / Molecular Weight (g/mol)) × 1000

= (4.0 g / 20000 g/mol) × 1000 = 0.2 mmol

The number of mmol of the peptide:

mmol of peptide = 8 × 0.2 mmol = 1.6 mmol

The theoretical weight of the peptide, assuming 100% yield:

1.6 mmol × 4206 g/mol (MW of peptide) = 6.7269 g

Yield Calculation Considering Losses

To determine the maximum achievable yield, the losses incurred during each coupling cycle were considered:

1. Initial mmol of peptide = 100%
2. Number of diafiltration steps per cycle = 10
3. Loss per diafiltration step due to 99.9% rejection = 0.1%
4. Number of coupling cycles = 10
5. Sampling loss per coupling cycle = 0.025% (10 µL from a total of 40 mL)
6. Total loss per coupling cycle = (0.1% × 10) + 0.025% = 1.025%

The yield after one coupling cycle= 100% - 1.025% = 98.975%

The yield after ten coupling cycles= (98.975%) ^ 10 = 90.2%

Thus, the maximum theoretical weight achievable= 90.2% × 6.7269 g = 6.067 g

Experimental Yield

After draining the synthesizer, 11.8 g of peptide-bound nanostar was obtained. For simplicity, 1 g was used for global deprotection, yielding 455 mg of isolated 39-mer peptide.

The total estimated peptide weight for the whole synthesis= 11.8 g × 455 mg / 1000= 5.369 g

The 39-mer peptide yield% = (5.369 g / 6.067 g) × 100% = 88%

5. NMR spectra

^{1}H NMR spectrum of the photolabile linker **4**.

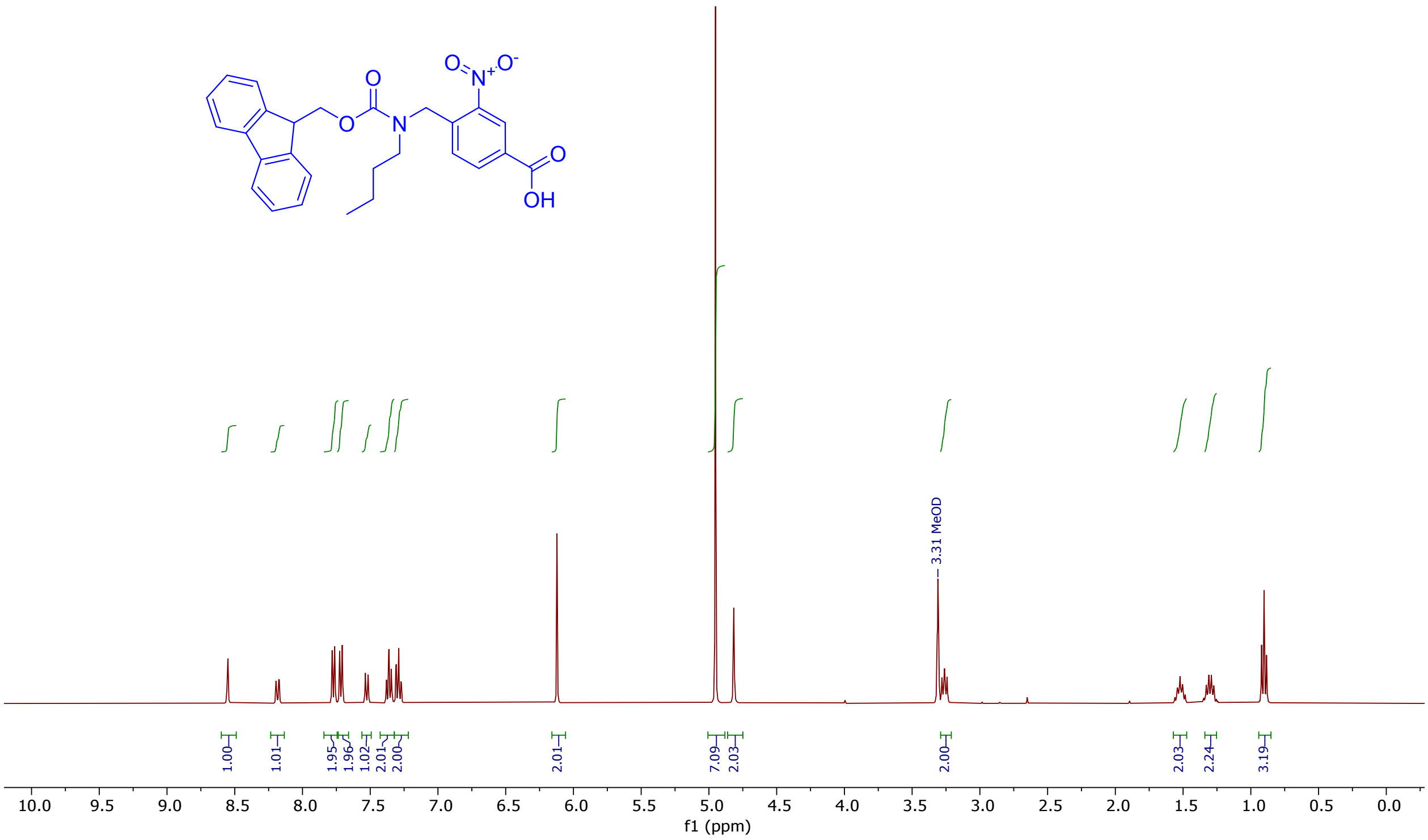

^{13}C NMR spectrum of the photolabile linker **4**.

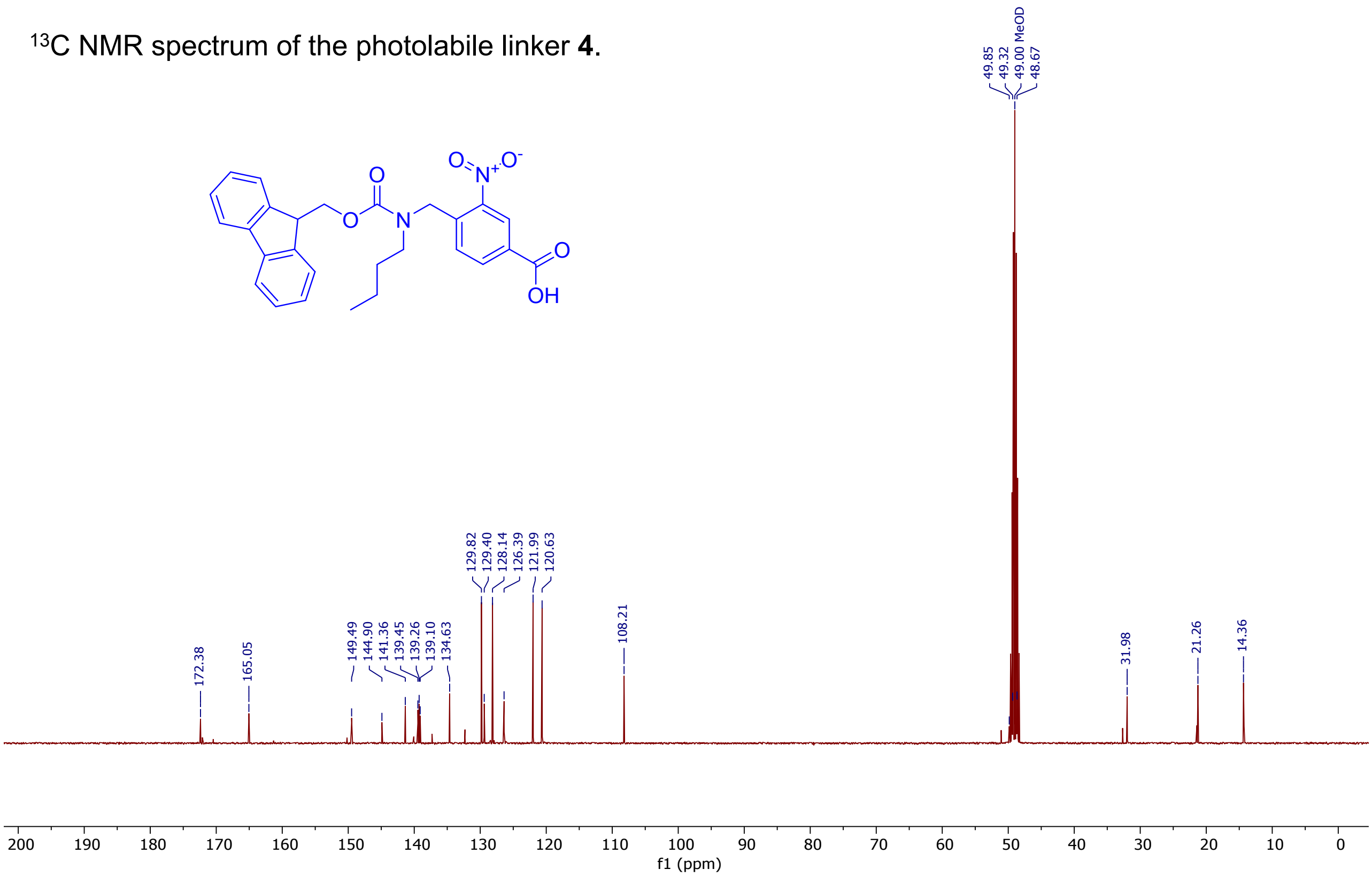

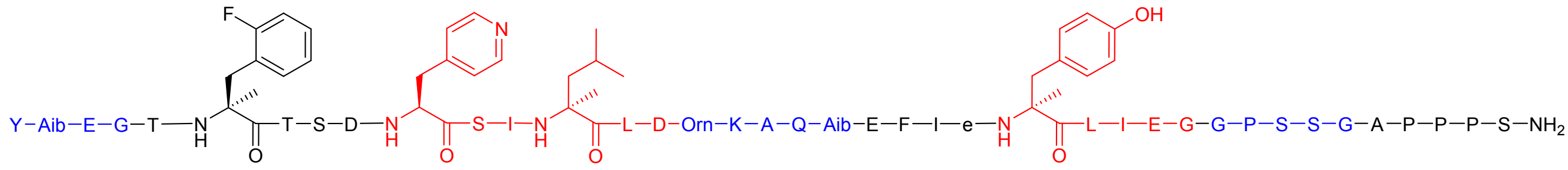

^{1}H NMR spectrum of the isolated peptide **9**.

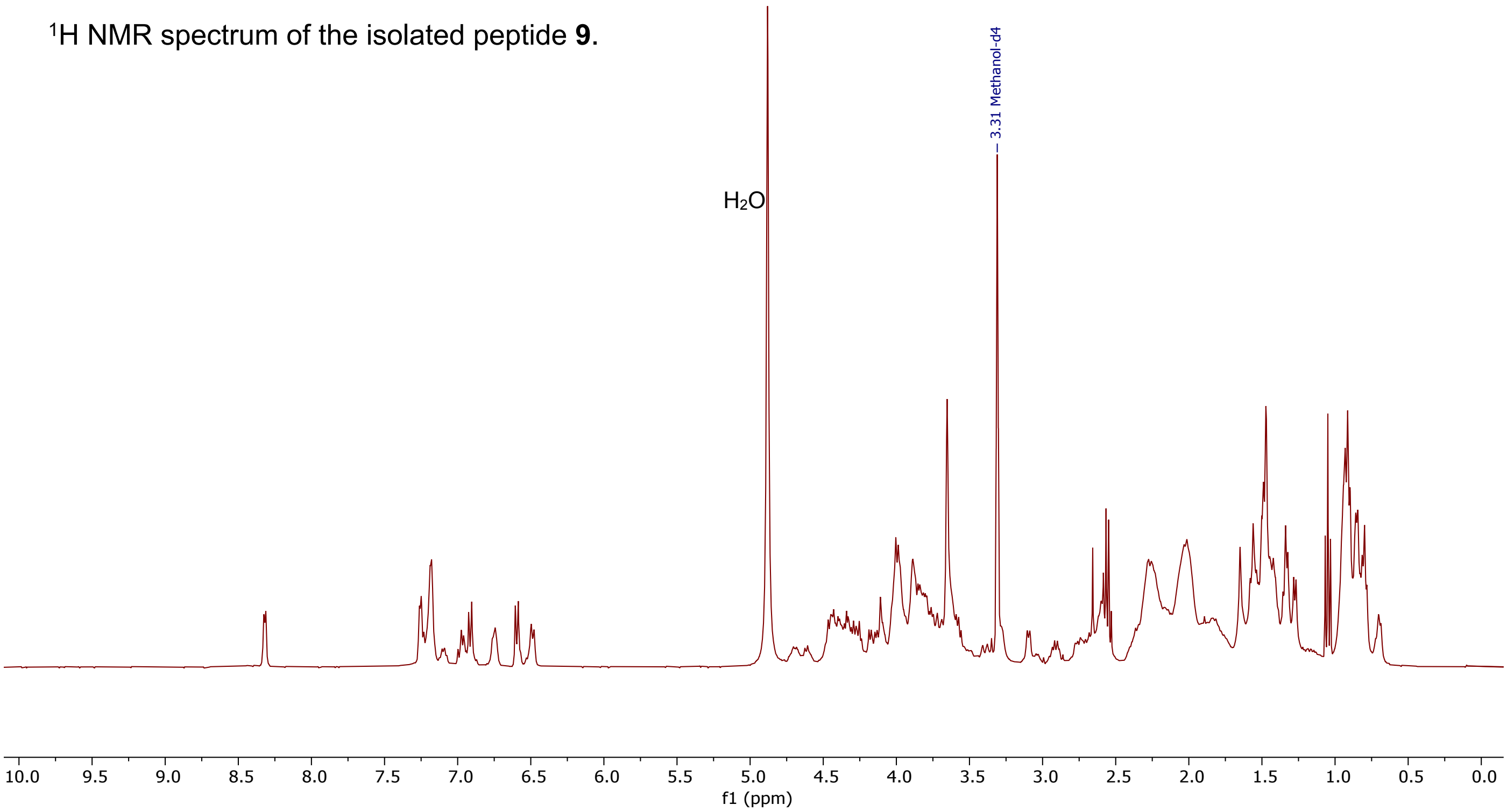

¹³C NMR spectrum of the isolated peptide **9**.

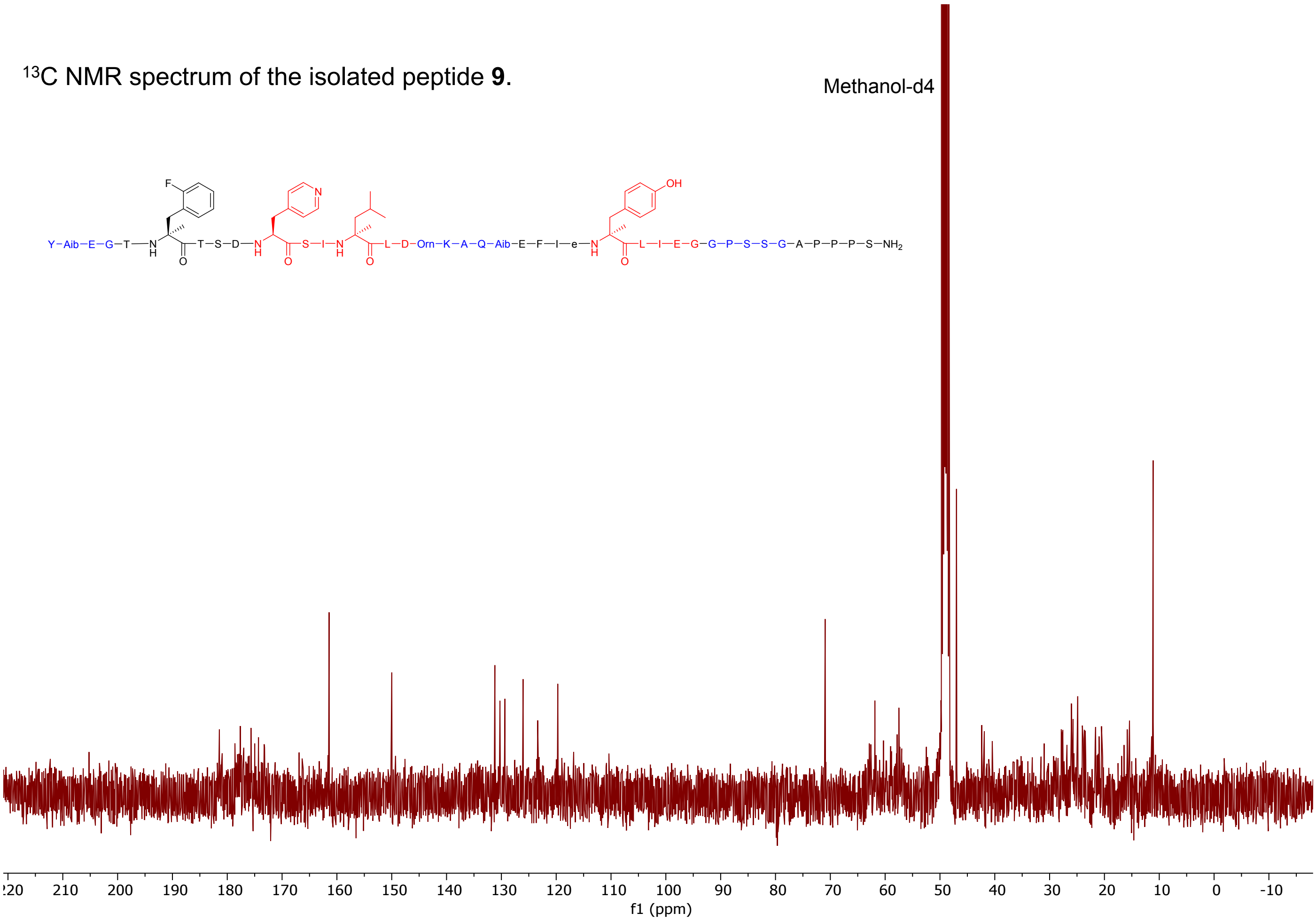

^{1}H NMR spectrum of the protected peptide-bound nanostar **S5**.

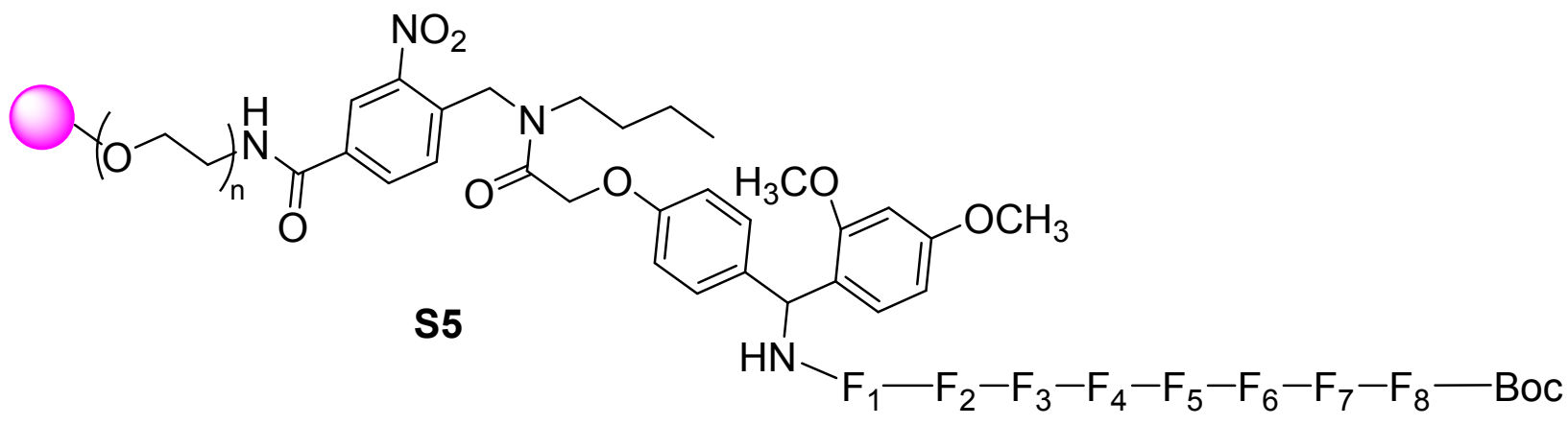

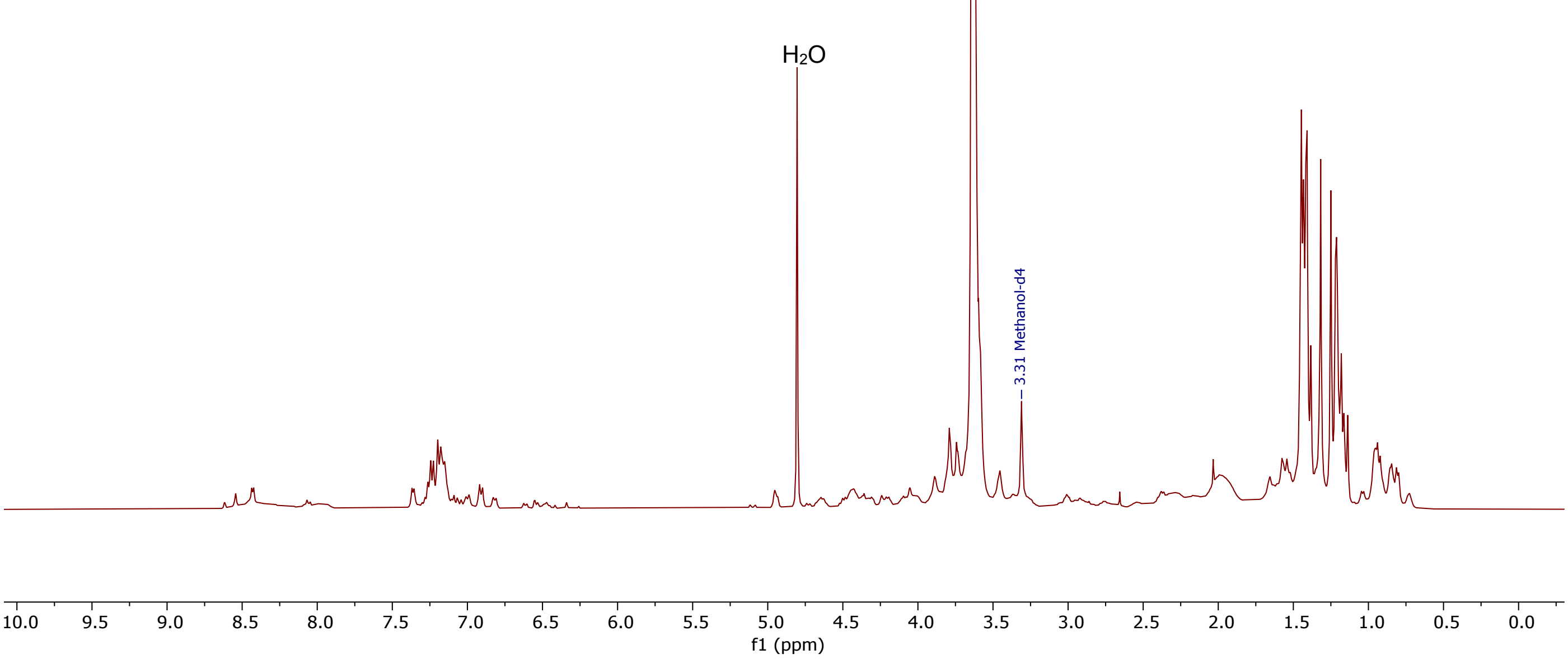

^{13}C NMR spectrum of the protected peptide-bound nanostar **S5**.

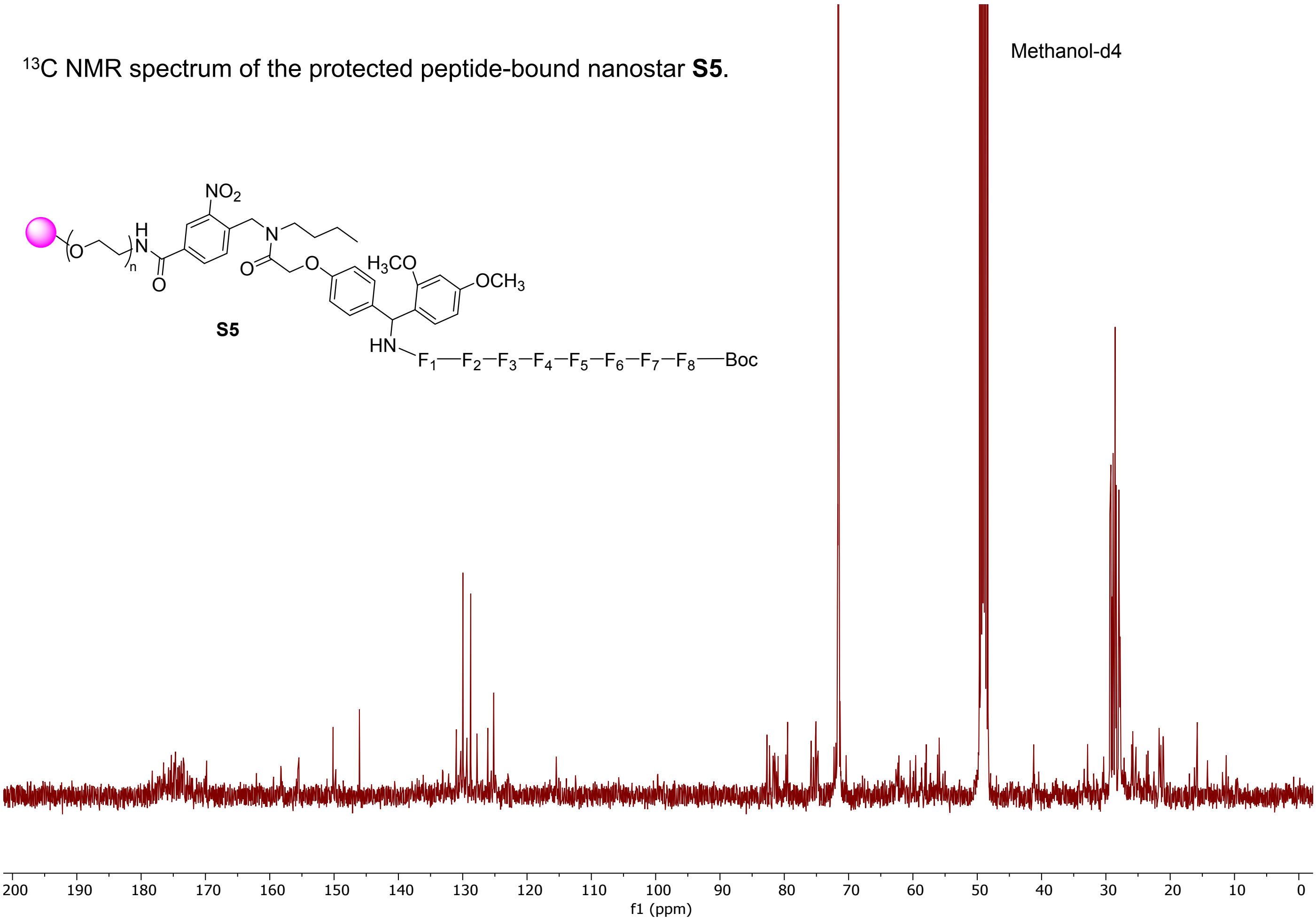

Appendix

The chemical structure and the exact mass of the 39-mer deprotected peptide **9**.

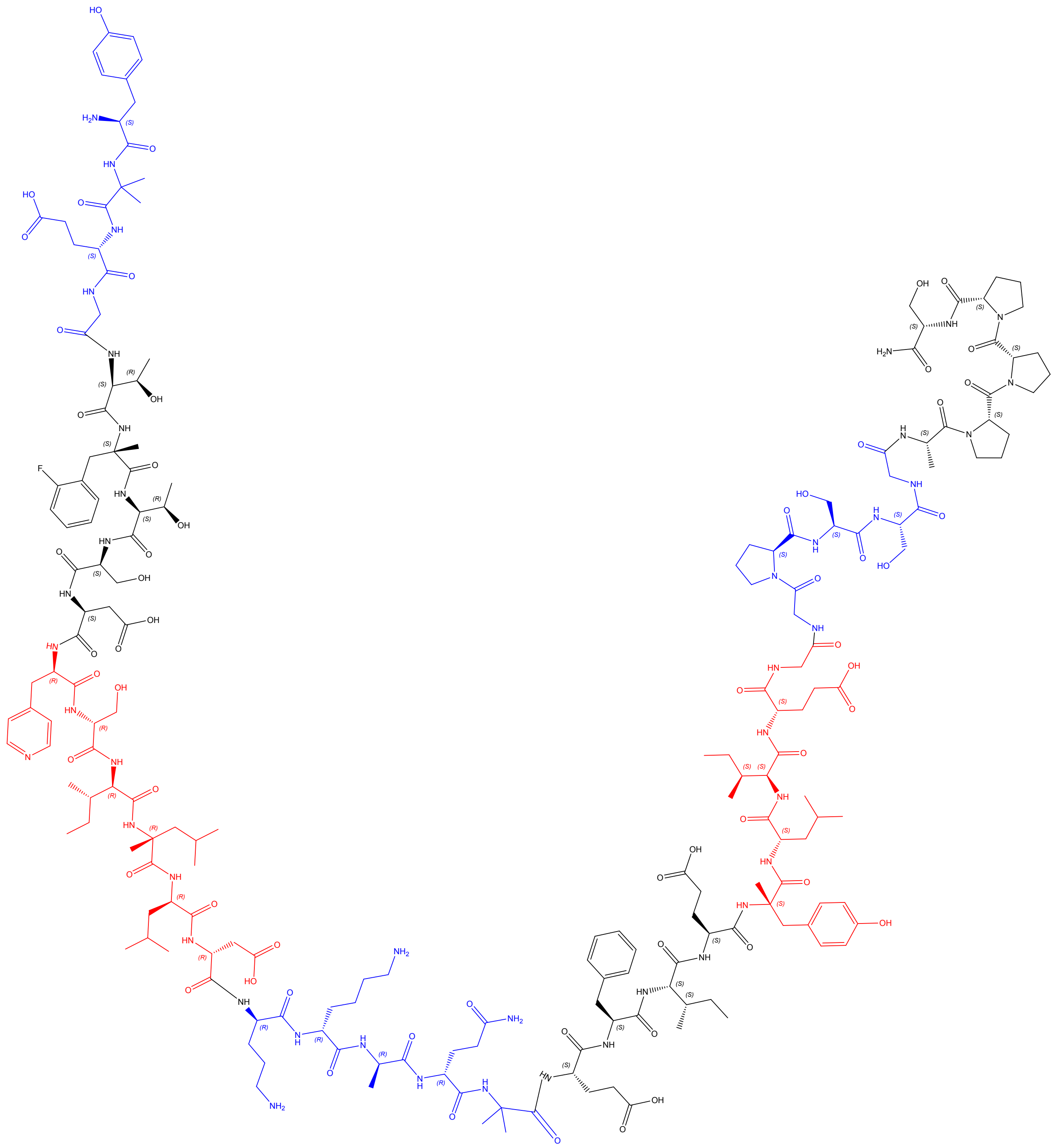

Exact Mass: 4206.08

The chemical structures and the exact mass of the photocleaved products **S6**, **S8**, **S9**, **S10**, **S11**, **S12**, and **S13**.

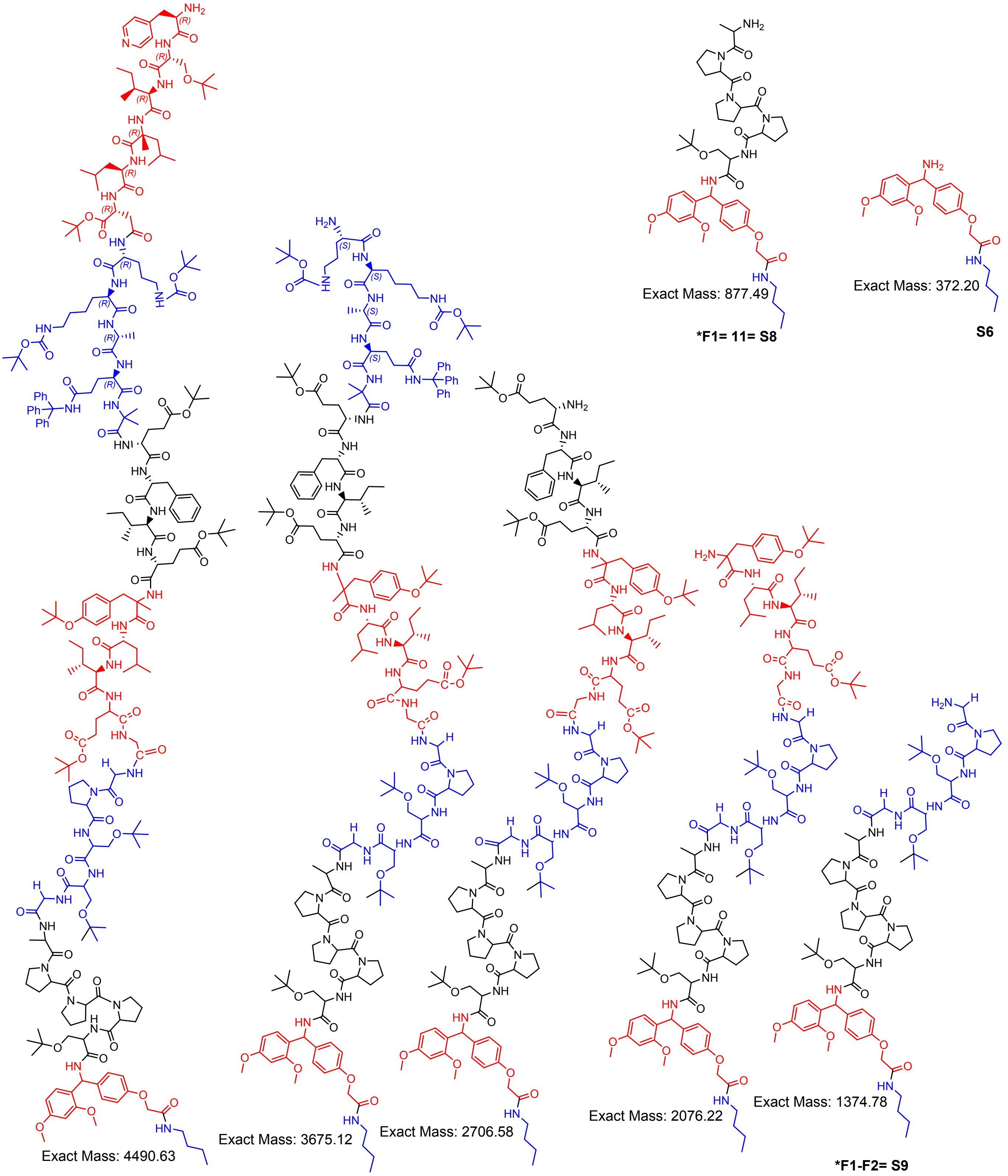

Exact Mass: 877.49

***F1= 11= S8**

Exact Mass: 372.20

S6

Exact Mass: 4490.63

***F1-F6= S13**

Exact Mass: 3675.12

***F1-F5= S12**

Exact Mass: 2706.58

***F1-F4= S11**

Exact Mass: 2076.22

***F1-F3 = S10**

Exact Mass: 1374.78

***F1-F2= S9**

The chemical structures and the exact mass of the photocleaved product **S14**.

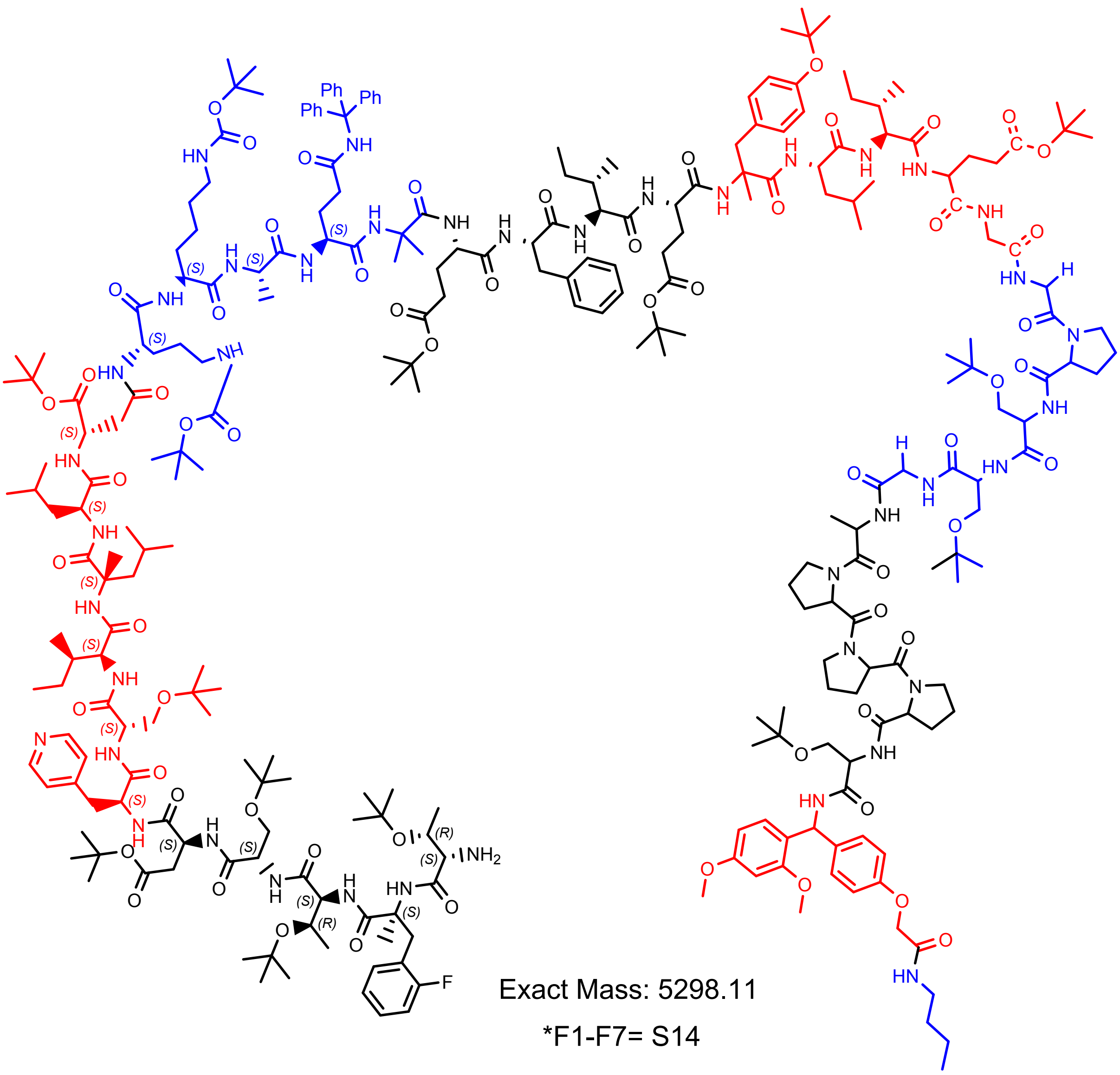

Exact Mass: 5298.11

*F1-F7= S14

The chemical structures and the exact mass of the photocleaved product **S15**.

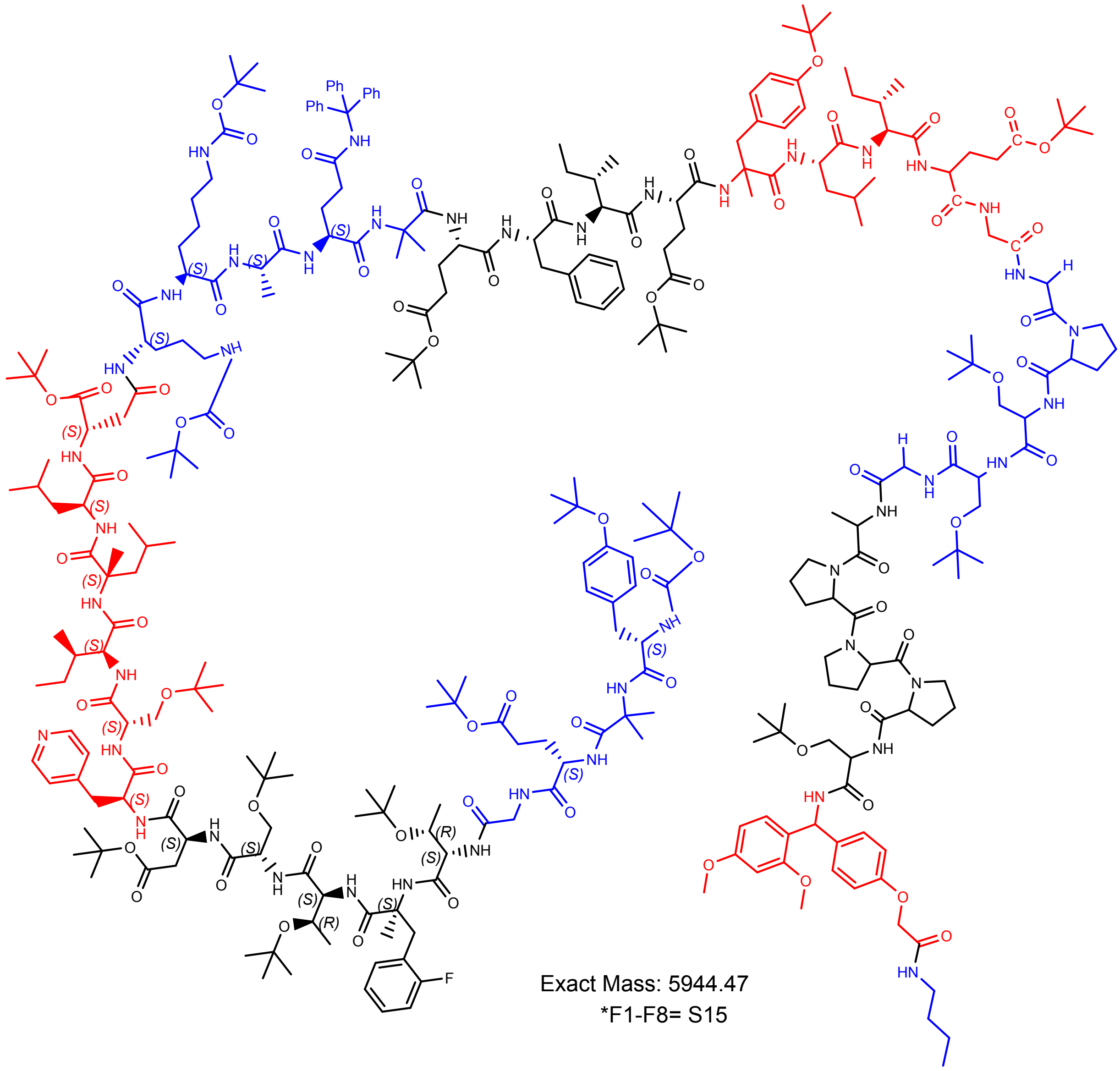

Supplement: Supplementary file 1 [file op6c00123_si_001.pdf]
